# The 2024 UK clinical guideline for the prevention and treatment of osteoporosis

**DOI:** 10.1007/s11657-025-01588-3

**Published:** 2025-09-08

**Authors:** Celia L. Gregson, David J. Armstrong, Christina Avgerinou, Jean Bowden, Cyrus Cooper, Lucy Douglas, John Edwards, Neil J. L. Gittoes, Nicholas C. Harvey, John A. Kanis, Sarah Leyland, Rebecca Low, Eugene McCloskey, Katie Moss, Jane Parker, Zoe Paskins, Kenneth Poole, David M. Reid, Mike Stone, Julia Tomson, Nic Vine, Juliet Compston, National Osteoporosis Guideline Group (NOGG)

**Affiliations:** 1 Musculoskeletal Research Unit, Bristol Medical School, Learning and Research Building, University of Bristol, Southmead Hospital, Bristol, BS10 5NB UK; 2 Royal United Hospital NHS Foundation Trust, Bath, UK; 3 Western Health and Social Care Trust (NI), Nutrition Innovation Centre for Food and Health, Ulster University, Belfast, UK; 4 Sterndale Surgery, London, UK; 5 Department of Primary Care and Population Health, University College London, London, UK; 6 MRC Lifecourse Epidemiology Centre, University of Southampton, Southampton, UK; 7 NIHR Southampton Biomedical Research Centre, University of Southampton and University Hospital Southampton NHS Foundation Trust, Southampton, UK; 8 NIHR Oxford Biomedical Research Centre, University of Oxford, Oxford, UK; 9 Lindley Group Practice Huddersfield, Huddersfield, UK; 10 Musculoskeletal Medicine and Rheumatology, East Lancashire Hospitals NHS Trust, Blackburn, UK; 11 Primary Care Centre Versus Arthritis, School of Medicine, Keele University, Keele, Staffordshire UK; 12 Wolstanton Medical Centre, Newcastle-Under-Lyme, UK; 13 Centre for Endocrinology, Diabetes and Metabolism, Queen Elizabeth Hospital, University Hospitals Birmingham & University of Birmingham, Birmingham, UK; 14 Centre for Metabolic Bone Diseases, University of Sheffield, Sheffield, UK; 15 Royal Osteoporosis Society, Bath, UK; 16 Marcham Road Health Centre, Abingdon, UK; 17 Metabolic Bone Disease, Nuffield Orthopaedic Centre, Oxford, UK; 18 Department of Oncology & Metabolism, MRC Versus Arthritis Centre for Integrated Research in Musculoskeletal Ageing (CIMA), Mellanby Centre for Musculoskeletal Research, University of Sheffield, Sheffield, UK; 19 St. George’s University Hospital, London, UK; 20 School of Medicine, Keele University, Haywood Academic Rheumatology Centre, Haywood Hospital, Midlands Partnership NHS Foundation Trust, Keele, Stoke-On-Trent UK; 21 Department of Medicine, University of Cambridge, Cambridge, UK; 22 University of Aberdeen, Aberdeen, Scotland; 23 University Hospital Llandough, Cardiff and Vale University Health Board, Llandough, UK; 24 School of Clinical Medicine, University of Cambridge, Cambridge, UK; 25 West Midlands, Birmingham, UK; 26 Preston, Lancashire UK; 27 Plymouth, Devon UK

**Keywords:** Osteoporosis, Fracture, NOGG, Guideline

## Abstract

**Summary:**

The National Osteoporosis Guideline Group (NOGG) has updated the revised UK guideline for the assessment and management of osteoporosis and the prevention of fragility fractures in postmenopausal women, and men age 50 years and older. This guideline is relevant for all healthcare professionals involved in osteoporosis management.

**Introduction:**

The UK National Osteoporosis Guideline Group (NOGG) first produced a guideline on the prevention and treatment of osteoporosis in 2008, with updates in 2013, 2017 and 2021. This paper presents a minor update of the 2021 guideline, the scope of which is to review the assessment and management of osteoporosis and the prevention of fragility fractures in postmenopausal women and men aged 50 years and older.

**Methods:**

Where available, systematic reviews, meta-analyses and randomised controlled trials have been used to provide the evidence base. Conclusions and recommendations have been systematically graded according to the strength of the available evidence.

**Results:**

Review of the evidence and recommendations are provided for the diagnosis of osteoporosis, fracture-risk assessment and intervention thresholds, management of vertebral fractures, non-pharmacological and pharmacological treatments, including duration and monitoring of anti-resorptive therapy, glucocorticoid-induced osteoporosis, as well as models of care for fracture prevention. Recommendations are made for training, service leads and commissioners of healthcare, and for review criteria for audit and quality improvement. Specific 2024 updates include guidance on fracture risk assessment by ethnicity, Parkinson’s disease, Down’s syndrome and lower-limb amputation; furthermore, the definition of very high fracture risk has been clarified. Hormone replacement therapy (HRT) is now recommended as a first-line treatment option in younger postmenopausal women with high fracture risk and low baseline risk for adverse events; recommendations regarding abaloparatide are included; additional training resources have been added.

**Conclusion:**

The guideline provides a comprehensive overview of the assessment and management of osteoporosis for all healthcare professionals involved in its management. This position paper has been endorsed by the International Osteoporosis Foundation and the European Society for Clinical and Economic Aspects of Osteoporosis, Osteoarthritis and Musculoskeletal Diseases (ESCEO).

## Introduction

This guideline was prepared in 2021 with the support of the societies listed to provide guidance on prevention and treatment of osteoporosis with the overarching aim of reducing fragility fracture risk, and updated in 2024, replacing previous National Osteoporosis Guideline Group (NOGG) guidance [1–4]. The scope of the guideline is to review the assessment and diagnosis of osteoporosis, the therapeutic interventions available and the approaches for the prevention of fragility fractures, in postmenopausal women, and in men aged 50 years or older. This focus is chosen as fragility fractures and osteoporosis are uncommon in premenopausal women and men younger than 50 years, and therefore, when these occur, patients need thorough investigation for secondary causes of osteoporosis, and careful consideration of treatment options. Specialist referral is usually required.

This NOGG guidance has appraised the current evidence base to inform these updated recommendations. The aim of the guideline is to provide clinically appropriate recommendations that integrate available evidence on clinical efficacy, effectiveness, and safety. This contrasts with, but complements, the remit of the National Institute for Health and Care Excellence (NICE), which focuses principally on establishing criteria for cost effectiveness. Cost effectiveness analyses are generally supportive for treatment guided by clinical effectiveness thresholds, rather than defining intervention thresholds per se [5]. The guideline has been prepared by a writing group and has been approved after consultation with stakeholders (Appendix 1).

The guideline is intended for all healthcare professionals involved in the prevention and treatment of osteoporosis and fragility fractures. This includes primary care practitioners, allied health professionals and relevant specialists in secondary care including rheumatologists, gerontologists, gynaecologists, endocrinologists, clinical biochemists, and orthopaedic surgeons. The guideline includes recommendations for training in osteoporosis care. The guideline is supported by a series of Frequently Asked Questions (FAQs) available on the NOGG website (https://www.nogg.org.uk/). The conclusions and recommendations in the document are systematically graded, according to the quality of information available, to indicate the level of evidence on which recommendations are based. The grading methodology is summarised in Appendix 2. Where available, systematic reviews, meta-analyses and randomised controlled trials have been used to provide the evidence base. The evidence base comprises systematic reviews and meta-analyses identified in PubMed from July 2016 to Sept 2020, with additional evidence added based on expertise and knowledge of the advances in the field, from within the group. The quality of systematic reviews and meta-analyses used in the formulation of recommendations was assessed using AMSTAR2 (Appendix 3). The updates to recommendations in this guideline were agreed by the National Osteoporosis Guideline Development Group.

This guideline provides a framework from which local management protocols should be developed to provide advice for healthcare professionals. Implementation of this guideline should be audited at a local and national level. The recommendations in the guideline should be used to aid management decisions but do not replace the need for clinical judgment in the care of individual patients in clinical practice.

## Summary of the 2024 update to the 2021 guideline

This revised guideline now includes clarification that quantitative ultrasound, IBEX bone health, and Radiofrequency Echographic Multi Spectrometry are not recommended for the diagnosis of osteoporosis; advice on the use of FRAX in non-white ethnic groups and those who have migrated to the UK; advice on FRAX adjustment when assessing patients with Parkinson’s disease; addition of clinical risk factors that increase fracture risk, specifically lower limb amputation and adult learning disabilities: e.g., Down’s Syndrome; the definition of very high fracture risk has been clarified; the intervention threshold has been defined and the associated NOGG graphs updated; vaping with e-cigarettes has been added as a possible risk factor for fragility fracture; hormone replacement therapy (HRT) is now recommended as a first-line treatment option in younger postmenopausal women (age ≤ 60 years) with high fracture risk and low baseline risk for adverse malignant and thromboembolic events; recommendations pertaining to the use of abaloparatide have been added; considerations when choosing which anabolic agent to use have been outlined; and additional training resources have been added.

## Background

The conceptual definition of osteoporosis was made by the World Health Organization (WHO) in 1994 as a ‘progressive systemic skeletal disease characterized by low bone mass and microarchitectural deterioration of bone tissue, with a consequent increase in bone fragility and susceptibility to fracture’ [6]. Since microarchitectural deterioration could not be measured clinically, the operational description was based on a bone mineral density (BMD) *T*-score of ≤ −2.5. Over the years this was adopted as a clinical definition; however, the limitations of focusing on a BMD-based definition alone have since become clear. BMD is now viewed as one, albeit very important, risk factor to be considered when assessing fracture risk, which is now viewed as the principal necessity.

The clinical significance of osteoporosis lies in the fractures that arise. In adults, approximately one in two women and one in five men will sustain one or more fragility fractures (a low trauma fracture sustained from a fall from standing height or less) in their lifetime [7]. In the UK, the prevalence of femoral neck BMD *T*-score ≤ −2.5, in those aged 50 years and older, is 6.8% in men and 21.8% in women [8]. However, the majority of people who sustain a fragility fracture will have a femoral neck BMD *T*-score above −2.5, reflecting the contribution of many other factors, besides BMD, to fracture risk [9–11]. Fall-related risk factors add significantly to fracture risk and often overlap with risk factors for osteoporosis, hence the need for integrated fall and fracture services.

Currently in the UK, approximately 549,000 new fragility fractures occur each year, including 105,000 hip fractures, 86,000 vertebral fractures, and 358,000 other fractures (i.e., fractures of the pelvis, ribs, humerus, forearm, tibia, fibula, clavicle, scapula, sternum, and other femoral fractures); 33% are sustained by men [8, 12, 13]. Such fractures cause severe pain, disability, reduction in quality of life, and in some cases increased mortality [14, 15]. In the UK, fragility fractures are estimated to account for 579,722 DALYs (Disability Adjusted Life Years) lost, largely driven by years lived with disability. This equates to 24 DALYs per 1000 people aged over 50 years, which is comparable to the DALYs lost from dementia [8]. Costs of fragility fractures to the National Health Service (NHS) exceed £4.7 billion per annum, of which £2.6 billion is directly incurred after an incident fracture (£1.1 billion for hip fractures alone [16]), with more than £1.7 billion attributable to institutional care costs post-fracture (estimated for 2017) [8]. Total direct costs for 2019 were £5.4 billion, accounting for 2.4% of healthcare spending [17].

Common sites of fragility fracture include the vertebral bodies, hip, distal radius, proximal humerus, and pelvis. Hip fracture is the most common reason for emergency anaesthesia and surgery in older people. It is also the most common cause of death following a fall. After hip fracture, the mean hospital length of stay is 20 days, accounting for half a million hospital bed days used each year, with 3600 hospital beds (3159 in England, 325 in Wales and 133 in Northern Ireland) occupied at any one time by patients recovering from hip fracture [18, 19]. Loss of independence is common following a hip fracture, with only 52% living in their own home after 120 days [12] and 26% will die within 12 months of their fracture [20]. Most major osteoporotic fractures are associated with reduced relative survival, part causally related and part due to associated co-morbidity [21–23].

In the UK, fracture rates vary by geographic location, race and levels of socioeconomic deprivation [24–26]. As in many higher income countries, age- and sex-adjusted fracture rates appear relatively stable, although increases in hip fractures amongst men in the UK have been reported [24, 27]. Changes in vertebral fracture rates potentially reflect secular alterations to reporting of cases. Importantly, the ageing of the UK population is predicted to give rise to a 19.6% increase in the number of fragility fractures by 2030 if changes are not made to current practice [8].

## Fracture risk assessment and case finding

### Recommendations

1. A FRAX assessment should be performed in any postmenopausal woman or man age ≥ 50 years, with a clinical risk factor for fragility fracture to guide BMD measurement and prompt timely referral and/or drug treatment where indicated (Strong recommendation).
2. When using FRAX to calculate the probability of fracture, clinical judgement is needed when clinical risk exceeds those factors able to be entered into FRAX (Strong recommendation).
3. Arithmetic adjustments to FRAX probabilities of major osteoporotic fracture (MOF: clinical spine, hip, forearm or humerus) and hip fracture (see Table 1) can be used in clinical practice to take account of additional clinical risk factors, such as high dose glucocorticoid use, discordantly low lumbar spine BMD, type 2 diabetes, and a history of falls (Conditional recommendation).
4. Vertebral fracture assessment (VFA) is indicated in postmenopausal women and men age ≥ 50 years if there is a history of ≥ 4 cm height loss, kyphosis, recent or current long-term oral glucocorticoid therapy, a BMD *T*-score ≤  − 2.5 at either the spine or hip, or in cases of acute onset back pain with risk factors for osteoporosis (Strong recommendation).
5. *T*-scores in men and women derived from femoral neck BMD should use normative values for BMD derived from young healthy women from NHANES III (Strong recommendation).
6. DXA scan results should be reported within 3 weeks of the scan, by healthcare professionals with specific training in DXA interpretation, and in accordance with national and international reporting standards (Strong recommendation).
7. Patients with osteoporosis and/or a fragility fracture should be investigated for underlying causes, including the need for routine blood tests (Strong recommendation).
8. The use of quantitative ultrasound, IBEX bone health, and Radiofrequency Echographic Multi-Spectrometry are not recommended for the diagnosis of osteoporosis (Strong recommendation).
9. QCT-measured femoral neck areal BMD in postmenopausal women and men aged ≥ 50 years can be used for opportunistic diagnosis of osteoporosis and to inform individual treatment decisions using FRAX (Conditional recommendation).
10. Computer Aided Diagnostics (CAD) may be considered to improve standard reporting of CTs performed on postmenopausal women and men aged ≥ 50 years to improve opportunistic identification of vertebral fractures (Conditional recommendation).

**Table 1:** Approximate adjustments and considerations to probabilities of hip fracture and major osteoporotic fracture to aid the interpretation of FRAX

Risk variable	Adjustment to FRAX*	Access
Medium and high dose exposure to oral glucocorticoids	Medium doses (2.5–7.5 mg daily) are the assumed minimum requirement for FRAX calculation, and the unadjusted FRAX value is used. For high doses (> 7.5 mg daily), MOF probabilities are upward revised by about 15% and hip fracture probabilities by 20% ^¥^	Automatic adjustment available on FRAX website: http://www.nogg.org.uk/manual-data-entry. Kanis et al. (2011) [28]
Concurrent data on lumbar spine (LS) BMD	Increase or decrease the MOF probability by 10% of each rounded *T*-score difference between LS and FN (see Frequently Asked Questions | NOGG no.4 for worked example)*	Leslie et al. (2011); Johansson et al. (2014) [29, 30]
Trabecular bone score (TBS)	Increase MOF probability by 30% for each standard deviation (SD) decrease in TBS	TBS adjustment can be accessed from the UK FRAX website. McCloskey et al. (2016) [31]
Hip axis length (HAL)	Increase or decrease hip fracture probability by 30% for each SD difference in HAL	Leslie et al. (2016) [32]
Falls history	Increase MOF and hip fracture probability by 30% for a history of recurrent falls (≥ 2 falls in the last year)	Masud et al. (2011) [33]; Vandenput et al. [34]
Country of birth	Use FRAX model for country of birth since individuals retain the risk characteristics of their country of origin	Johansson et al. (2015) [35]; Wändell et al. (2021) [36]
Type 1 and 2 diabetes mellitus	Enter ‘yes’ in the rheumatoid arthritis input to FRAX	Other adjustments in Leslie et al. (2018) [37]
Parkinson’s disease, and related movement disorders	Enter ‘yes’ in the rheumatoid arthritis input to FRAX	Schini et al. (2023) [38]
Recent MOF	Marked uplift to fracture probabilities	*Kanis *et al*. (2020) *[39]

^*^Downward adjustment to FRAX probabilities should only be made in the context of a very reliable high lumbar spine BMD measurement and not on the basis of a discordant result due to artefact, e.g., from degenerative change

^¥^See ‘glucocorticoid-induced osteoporosis’ for further details on glucocorticoid doses and recommendations

### Measurement of bone mineral density

The risk of fracture increases progressively with decreasing bone mineral density (BMD). Systematic reviews and meta-analyses of observational population-based studies using absorptiometric techniques indicate that the risk of fracture increases approximately two-fold for each standard deviation (SD) decrease in BMD [40, 41]; (Evidence level Ia). The gradient of fracture risk varies according to the site and technique used, the person’s age, and the fracture type [41]; (Evidence level Ia). The predictive value of BMD for hip fracture is at least as good as that of blood pressure for stroke [42]; (Evidence level IV).

The WHO and the International Osteoporosis Foundation (IOF) recommend that the reference technology for the measurement of BMD is dual-energy X-ray absorptiometry (DXA) applied to the femoral neck, because of its higher predictive value for fracture [43, 44]; (Evidence level Ia). DXA measurements of femoral neck BMD are used in FRAX®. The spine is not always a reliable site for risk assessment or for the diagnosis of osteoporosis in older people because of the high prevalence of degenerative changes, which artefactually increase the BMD value. However, a result in an older person showing low BMD is almost always valid and clinically useful, particularly in those people with disproportionately low spine BMD compared to the hip, but may not always relate to osteoporosis (e.g. osteomalacia can also cause low BMD). At the same DXA-measured femoral neck BMD, men and women are at approximately the same fracture risk [45, 46]; (Evidence level IIa). Therefore, the recommended reference range, from which femoral neck and total hip *T*-scores are calculated for men, women and transgender individuals in the US, is that derived from the NHANES III survey for white women aged 20 to 29 years [44, 47]. The reference ranges, from which lumbar spine and distal forearm *T*-scores are calculated, for both men and women of all ethnicities, are usually those of the manufacturer of the DXA scanner [47].

Osteoporosis can be diagnosed on the basis of the BMD *T*-score measured at the total hip, femoral neck, or lumbar spine. However, fracture risk prediction is not improved by the use of measurements from multiple sites [48, 49]; (Evidence level IIa). Where hip BMD measurement is not possible for technical reasons, or if the spine is differentially affected, then spine BMD measurements can be used for diagnosis. A diagnosis of osteoporosis can be made based on distal forearm (1/3 radius) *T*-score if neither spine nor hip can be reliably measured or interpreted, or if a patient exceeds the weight limit for the DXA table [47]; (Evidence level IV). Serial BMD measurement can be used to monitor response to treatment [50]. Lumbar spine BMD shows the largest treatment-related changes and is the preferred site, although if spinal degenerative changes are marked, BMD at the hip is a better site for monitoring. The validity of BMD measurements depends on good quality control and national (Royal Osteoporosis Society) and international (International Society for Clinical Densitometry) bodies have published standards for the reporting of DXA scans [47, 51]. Derivation of DXA-measured BMD Z-Scores, which compare a BMD measure with average BMD of people of the same age, sex, and ethnicity, is recommended in men < 50 years and pre-menopausal women [47]. A low Z-Score can indicate the need for further investigation (see Table 2).

**Table 2:** Proposed clinical investigations to consider for the investigation of osteoporosis/fragility fractures

Routine	Other procedures, if indicated
Clinical history	Serum electrophoresis, serum immunoglobulins and serum free light chain assay
Physical examination including measurement of height and assessment of thoracic kyphosis	Plasma parathyroid hormone (PTH)^b^
Full blood cell count	Serum testosterone, sex hormone binding globulin, follicle stimulating hormone, luteinizing hormone
Erythrocyte sedimentation rate or C-reactive protein	24-h urinary free cortisol/overnight dexamethasone suppression test
Renal function	Serum prolactin
Serum calcium, albumin, creatinine, phosphate^a^, alkaline phosphatase^a^ and liver transaminases	Serum magnesium if hypocalcaemicTissue transglutaminase antibodies, +/− endomysial antibodies (coeliac disease screen)
Serum 25-hydroxyvitamin D	Urinary calcium excretion
Thyroid function tests	Markers of bone turnover (e.g., CTX, P1NP)^c^Lateral radiographs of lumbar and thoracic spine or DXA based lateral vertebral imagingBone densitometry (DXA) if indicated by FRAX assessment and/or required for BMD monitoringIsotope bone scan

^a^Persistent low phosphate or alkaline phosphatase should not be overlooked as this can indicate underlying metabolic bone disease

^b^Measure PTH if albumin-adjusted serum calcium ≥ 2.6 mmol/l twice, or if ≥ 2.5 mmol/l twice if primary hyperparathyroidism is suspected[52]

^c^Principally measured to monitor bone turnover in response to anti-resorptive treatment, CTX reflects bone resorption, P1NP reflects bone formation. CTX is best measured in the morning after an overnight fast. Other investigations—for example, bone biopsy and genetic testing for osteogenesis imperfecta—are largely restricted to specialist centres

QCT-measured femoral neck areal BMD predicts osteoporotic fractures in men and women and is equivalent to DXA-derived areal BMD [53–55]. Femoral neck and total hip *T*-scores calculated from two-dimensional projections of quantitative computed tomography (QCT) data are equivalent to the corresponding DXA-derived *T*-scores. Thus, femoral neck CT X-ray absorptiometry (CTXA) BMD measurements can be included in FRAX [47, 56–58]; (Evidence level IIa). Other techniques for assessing skeletal BMD, including quantitative ultrasound, have been less well validated than absorptiometric techniques.

### Assessment of clinical risk factors

The performance characteristics of BMD assessment can be improved by the concurrent consideration of clinical risk factors that operate independently of BMD. Of particular importance is age, which contributes to risk independently of BMD [11, 59]; (Evidence level Ia). Additional clinical risk factors have been identified that provide information on fracture risk independently of both age and BMD:

i. Low body mass index (BMI) is a significant risk factor for hip fracture, but the value of BMI in predicting other fractures is very much diminished when adjusted for BMD [60]; (Evidence level Ia).
ii. A history of a prior fracture, particularly if sustained from low trauma and at a site characteristic for osteoporosis, is an important risk factor for further fracture [61]. The risks are in part independent of BMD [62]. Fracture risk is approximately doubled in the presence of a prior fracture, including asymptomatic moderate or severe (Grade 2 or 3) morphometric vertebral fractures [62, 63]; (Evidence level Ia). The increase in risk is even more marked for more than one vertebral fracture. After a fracture, the risk of subsequent fracture is highest in the immediate post-fracture interval (imminent risk) with more than one-third of subsequent fractures over a ten-year time frame occurring within the first year [39, 64]; (Evidence level Ic).
iii. A parental history of hip fracture is a significant risk factor that is largely independent of BMD [65]; (Evidence level Ia).
iv. Smoking is a risk factor that is in part dependent on BMD [66]; (Evidence level Ia).
v. Oral glucocorticoid therapy increases fracture risk in a dose-dependent manner. The fracture risk conferred by the use of glucocorticoids is, however, not solely dependent upon bone loss and BMD-independent risks have been identified [67, 68]; (Evidence level Ia).
vi. Alcohol intake shows a dose-dependent relationship with fracture risk. Where alcohol intake is on average two units or less daily, no increase in risk has been identified. Intakes of 3 or more units daily are associated with a dose-dependent increase in fracture risk [69]; (Evidence level Ia).
vii. There are many secondary causes of osteoporosis (e.g., inflammatory bowel disease, endocrine disorders), but in most instances it is uncertain to what extent an increase in fracture risk is dependent on low BMD or other factors such as the use of glucocorticoids. By contrast, rheumatoid arthritis increases fracture risk independently of BMD and the use of glucocorticoids [68]; (Evidence level Ia).
viii. Diabetes mellitus (both type 1 and type 2) is associated with an increase in risk of hip and non-vertebral fracture. In type 2 diabetes, a longer duration of disease and insulin use are associated with an increased risk [70, 71]; (Evidence level Ia), which is partly independent of BMD [72, 73].

The use of combined clinical risk factors alone to predict fracture risk performs very similarly to that of BMD alone [74]. The use of clinical risk factors with the addition of BMD is optimal, but BMD measurement can be targeted to those close to the threshold of low/high risk or close to the threshold of high/very high risk. There are many additional clinical risk factors for fracture not included in FRAX, including risks that either act solely by reducing BMD or have been less well validated or identify a risk that may not be amenable to particular treatments [11, 75]. Liability to falls is an example of the latter, where the risk of fracture is high, and treatment with drugs affecting bone metabolism alone may not fully address this risk [76].

In addition to glucocorticoids, several medications are known to increase hip fracture risk, including thyroid hormone excess, aromatase inhibitors for the treatment of breast cancer, and androgen deprivation for the treatment of prostate cancer [77–81]; (Evidence level Ia). Thiazolidinediones, used in the treatment of type 2 diabetes, also increase fracture risk [82, 83].

Several other drugs have been associated with increased fracture risk including antidepressants, antiparkinsonian drugs, antipsychotic drugs, anxiolytic drugs, benzodiazepines, sedatives, H2 receptor antagonists, and proton pump inhibitors [77–81]. The extent to which fracture risk is mediated by low BMD, fall risks, or other factors, or indeed is definitely causal in each case, is not known and therefore no specific recommendation is made regarding cessation. The impact of sex steroids on bone health in transgender individuals is unclear [84]. Biochemical indices of skeletal turnover have the potential to aid risk assessment but probably play a more immediate role in the monitoring of treatment [85–87]; (Evidence level Ia).

### Fracture risk assessment tools

The IOF and the WHO recommend that the risk of fracture is expressed as an absolute risk, i.e., probability over a ten-year interval [11]. The absolute risk of fracture depends upon age and life expectancy as well as the current relative risk. The period of 10 years covers the likely initial duration of treatment and the benefits that may continue if treatment is stopped. Shorter time horizons do not aid the categorisation of risk [88, 89]. Where life expectancy is less than 10 years, FRAX provides the remaining lifetime probability of fracture. Algorithms that integrate the weight of clinical risk factors for fracture risk, with or without information on BMD, were developed in 2008 by the then WHO Collaborating Centre for Metabolic Bone Diseases at Sheffield. The FRAX tool (https://www.fraxplus.org/calculation-tool/) computes the 10-year probability of hip fracture and/or of major osteoporotic fracture. A major osteoporotic fracture is a clinical spine, hip, forearm or humerus fracture. The tool has been externally validated in independent cohorts [59, 90]; (Evidence level Ia).

QFracture is based on a UK prospective open cohort study of routinely collected data from general practices that takes into account numerous clinical risk factors and estimates the 1 to 10 year cumulative incidence of hip and/or major osteoporotic fracture (http://www.qfracture.org [91]). The NICE has recommended the use of fracture risk assessment tools (FRAX or QFracture) in the assessment of patients [92]. Since FRAX and QFracture yield different outputs (probability of fracture accounting for mortality risk in the case of FRAX, and a cumulative risk of fracture in the case of QFracture), the two calculators cannot be used interchangeably. In addition, BMD cannot be incorporated into QFracture estimations. Finally, the NOGG intervention thresholds, recommended by NICE Quality Standards, are based on FRAX probability and thus cannot be used with fracture risk derived from QFracture or other calculators [90, 93].

Whilst QFracture takes account of several ethnicities, the FRAX tool takes a different approach. Importantly, the UK FRAX is calibrated to a majority white population, with a small minority of other ethnic groups included, so that the generated probabilities reflect a predominantly white population. When using FRAX in individuals who have moved from another country to the UK, regardless of ethnicity, the use of the FRAX model for their country of birth, if it exists, is appropriate since individuals retain the risk characteristics of their country of birth [35] [36]. While fracture risk in second and subsequent generations might move towards that in the general UK population, the accuracy of this assumption and speed of transition is not known for either QFracture or FRAX [94].

The input into FRAX includes, with age and sex, BMD independent clinical risk factors including: Body mass index (calculated from weight and height in kg/m^2^), previous fragility fracture (including morphometric vertebral fracture), parental history of hip fracture, current glucocorticoid treatment (any dose, by mouth for 3 months or more), current smoking, alcohol intake of 3 or more units daily, rheumatoid arthritis, secondary causes of osteoporosis (including: type 1 diabetes, long-standing untreated hyperthyroidism, untreated hypogonadism/premature menopause (< 45 years), chronic malnutrition/malabsorption, chronic liver disease, non-dialysis chronic renal failure (i.e., CKD 3a – 5). Femoral neck BMD is an optional input. The listed secondary causes are conservatively assumed to be mediated through low BMD and carry no weight when femoral neck BMD is entered into FRAX. Femoral neck BMD is an optional input. The listed secondary causes are conservatively assumed to be mediated through low BMD and carry no weight when femoral neck BMD is entered into FRAX.

FRAX assessment takes no account of prior osteoporosis drug treatment, or of the dose of several clinical risk factors. For example, a history of two prior fractures carries a higher risk than a single prior fracture. A prior clinical vertebral fracture carries an approximately two-fold higher risk than other prior fracture types. Dose responses are also evident for glucocorticoid use and are partially addressed in the NOGG guideline. Since it is not possible to model all such scenarios within the FRAX algorithm, clinical judgement is needed to interpret FRAX outputs.

High and low impact injuries exist on a continuum and the clinical significance of high and low impact fractures is blurred in the context of osteoporosis. Indeed, prior high-trauma fractures are associated with low BMD and future fracture risk to the same extent as fractures without high-trauma [61]. Although FRAX has a limited input of variables, relatively simple arithmetic procedures are available (Table 1) which can be applied to conventional FRAX estimates of probabilities of hip fracture and major osteoporotic fracture to adjust the probability assessment with knowledge of: high, moderate and low exposure to oral glucocorticoids [28]; (Evidence level IIa), concurrent data on lumbar spine BMD [29, 30]; (Evidence level Ia), information on trabecular bone score (TBS) [31]; (Evidence level Ia). TBS values can be entered on the UK FRAX website, hip axis length [32]; (Evidence level Ib), falls history [33]; (Evidence level IIa), country of birth [35]; (Evidence level Ib), type 2 diabetes mellitus [37]; (Evidence level Ib), Parkinson’s disease [38]; (Evidence level Ic), and recent major osteoporotic fracture (MOF) [39]; (Evidence level Ib). When applying these FRAX adjustments, a suggested increase of *x*% should be applied as a proportion of the original FRAX score. For example, uplifting the FRAX probability of 30% by 10% gives an adjusted probability of 30 × 1.10 = 33%. There is no evidence base available to inform on the accuracy of multiple adjustments. Pragmatically, the adjustment should be made for the most dominant factor, i.e. that which will have the greater impact on the estimated probability; (Evidence level IV). Although type 1 diabetes carries a risk of fracture over and above that provided by FRAX, there are yet no empirical data from which to recommend adjustment. In the meanwhile, adjustment can be used as for type 2 diabetes: (Evidence level IV). Parkinson’s disease (PD), and related movement disorders substantially increase both hip and non-vertebral fracture risk, independent of BMD [38, 95]. Entering PD as a risk variable using the rheumatoid arthritis input as a surrogate only partly accounts for this increased fracture risk [38]; (Evidence level Ib). Additionally, FRAX values have been shown to be largely unaffected by socioeconomic status [96], variation in body composition [97], cancer [98] and chronic renal disease [99]; (Evidence level Ib). Adjustments to FRAX probabilities which take into account severity and/or number of vertebral fractures can be made using the https://www.fraxplus.org/calculation-tool; however, currently payment is needed for each calculation.

Risk is best presented to patients numerically using simple frequencies and positive and negative framing e.g., for a 23% risk say ‘100 people like you, over the next 10 years, 23 will break a bone and 77 will not’. Describing risks solely with words, such as ‘You have a *high* chance of experiencing a fracture’ is ineffective and does not provide patients with the details needed to make an informed decision; it increases risk perceptions, and patients vary in their interpretations of what are low and high risks. It is easier for patients to understand whole numbers and simple frequencies (e.g., 1 in 100) rather than percentages. Graphs and pictograms make numeric information easier to understand and should be used where available [100]; (Evidence level IV).

### Investigation of osteoporosis and fragility fractures

Diagnostic assessment of individuals with osteoporosis should exclude diseases that mimic osteoporosis, identify the cause(s) of the osteoporosis, and include the management of any associated comorbidity. Common investigations are given in Table 2.

### Vertebral fracture assessment

The majority of vertebral fractures do not currently come to medical attention and thus remain undiagnosed [101]. Moderate or severe vertebral fractures, even when asymptomatic, are strong risk factors for subsequent fracture at the spine and other skeletal sites [63, 102, 103]; (Evidence level Ia). Vertebral fracture assessment (VFA) should therefore be considered in high-risk individuals, using either lateral lumbar and thoracic spine radiographs or lateral spine DXA imaging [104]; (Evidence level Ia). The latter delivers a significantly lower radiation dose whilst performing comparably to traditional radiographs [105]. Identification of vertebral fractures on routine radiological images, such as plain abdominal and chest radiographs, performed for other indications, offers the opportunity to detect clinically important osteoporotic fractures. Opportunistic diagnosis of osteoporosis and vertebral fractures is feasible using CT scans acquired for various clinical reasons, since the hip and spine are frequently in the scan field [106]; (Evidence level Ia). Vertebral fracture identification from CT using Computer Aided Diagnostics (CAD) can augment and improve standard reporting methods [107–110]; (Evidence level IIb). Reliable CAD methods have high sensitivity, specificity, and accuracy for vertebral fracture detection; (Evidence level IV).

### Screening and case finding

At present, there is no universally accepted policy for population-based screening to identify people with osteoporosis. With the recognition that factors in addition to BMD can improve fracture risk prediction, it is possible that screening strategies might be implemented in the future. A trial of screening in the UK used FRAX to target osteoporosis drug treatment to women at high risk of hip fracture. The risk assessment, with subsequent femoral neck BMD measurement and input to FRAX in intermediate/high risk individuals, was conducted in a primary care setting and involved almost 12,500 women aged 70–85 years. Over 5 years, compared to standard clinical care, the screening programme reduced the number of hip fractures by 28%. Similar results were observed in a study from Denmark [111], but with lesser effects observed in a further study in the Netherlands [112]. A meta-analysis of the three trials showed that screening reduced hip fracture risk by 20% [113]; (Evidence level Ia).

In the absence of a screening policy, a case-finding strategy is appropriate where patients are identified because of a fragility fracture or by the presence of other clinical risk factors. There are many clinical risk factors for fracture in addition to those included in FRAX which can be used to trigger fracture risk assessment (see Table 3), including thoracic kyphosis and height loss (≥ 4 cm), either in comparison with recalled young adult height or a documented loss on serial measurements [114]; (Evidence level IIa), and bariatric surgery resulting in malabsorption [115]; (Evidence level Ia).

**Table 3:** Clinical risk factors for osteoporosis/fractures, not accommodated in FRAX, which should trigger fracture risk assessment

Thoracic kyphosis
Height loss (≥ 4 cm)
Falls and frailty
Inflammatory disease: e.g., ankylosing spondylitis, other inflammatory arthritides, connective tissue diseases, systemic lupus erythematosus
Endocrine disease: e.g., Type 1 and 2 diabetes mellitus^a^, hyperparathyroidism, hyperthyroidism, hypogonadism, Cushing’s disease/syndrome
Haematological disorders/malignancy e.g., multiple myeloma, thalassaemia
Muscle disease: e.g., myositis, myopathies and dystrophies, sarcopenia
Lower limb amputation
Lung disease: e.g., asthma, cystic fibrosis, chronic obstructive pulmonary disease
HIV
Neurological/psychiatric disease e.g., Parkinson’s disease and associated syndromes ^a^, multiple sclerosis, epilepsy, stroke, depression, dementia
Adult learning disabilities: e.g., Down’s Syndrome
Nutritional deficiencies: calcium, vitamin D [note that vitamin D deficiency may contribute to fracture risk through undermineralisation of bone (osteomalacia) rather than osteoporosis]
Bariatric surgery and other conditions associated with intestinal malabsorption; e.g., inflammatory bowel disease, celiac disease
Medications, e.g.:
Some immunosuppressants (calmodulin/calcineurine phosphatase inhibitors)
(Excess) thyroid hormone treatment (levothyroxine and/or liothyronine). Patients with thyroid cancer with suppressed TSH are at particular risk
Drugs affecting gonadal hormone production (aromatase inhibitors, androgen deprivation therapy, medroxyprogesterone acetate, gonadotrophin hormone releasing agonists, gonadotrophin hormone receptor antagonists)
Some diabetes drugs (e.g., thiazolidinediones)
Some antiepileptics (e.g., phenytoin and carbamazepine)

^a^Able to be accommodated in FRAX by proxy, by entering ‘yes’ in the rheumatoid arthritis input (see Table 2)

## Intervention thresholds and strategy

### Recommendations

**Fig. 1:**
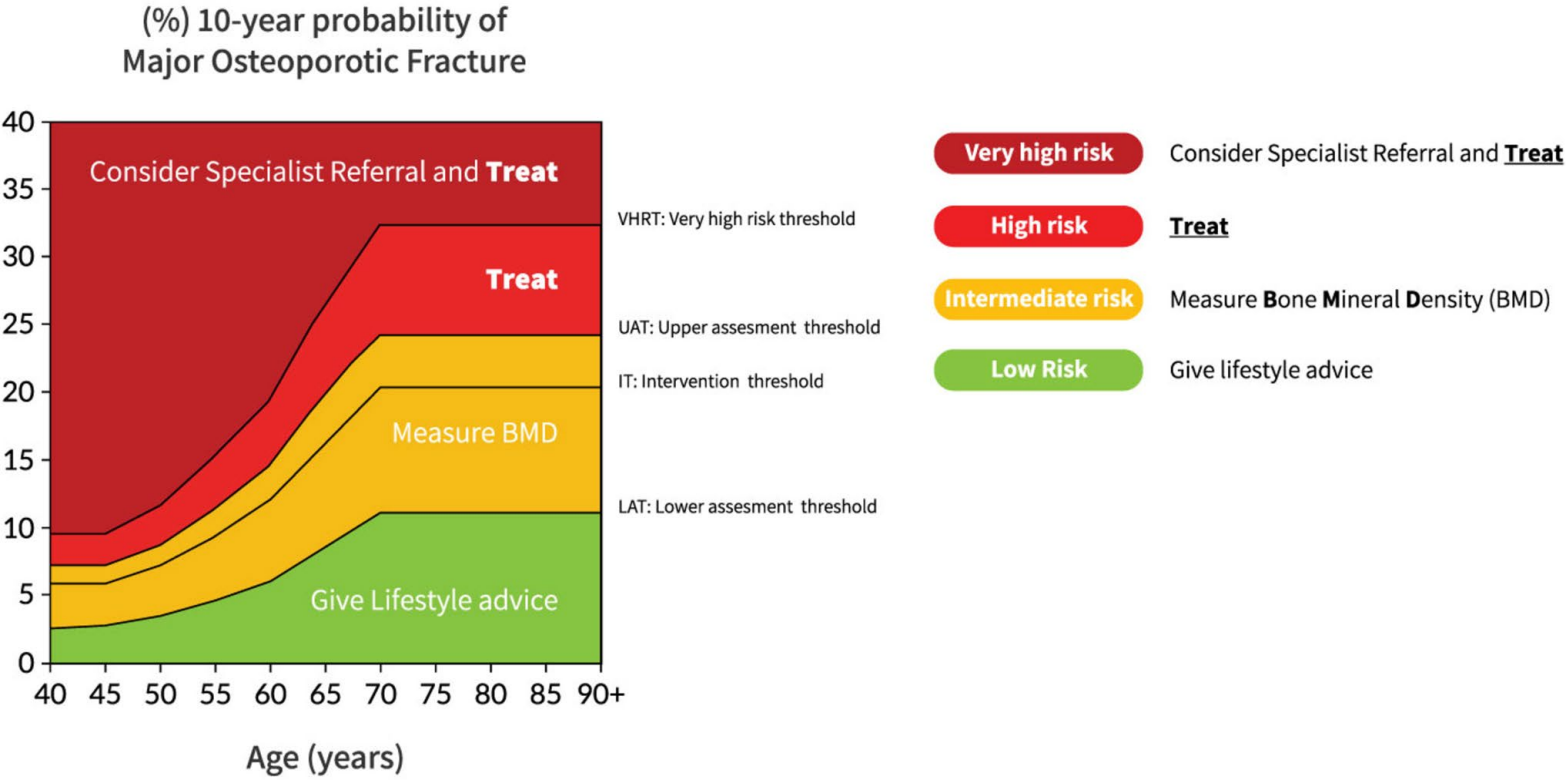
NOGG assessment, interventions, and risk thresholds for major osteoporotic fracture probability (MOF) in the UK with the use of FRAX. Individuals with probabilities below the lower assessment threshold (LAT) are considered for lifestyle advice. Those at intermediate risk (probabilities between the upper assessment threshold (UAT) and lower assessment threshold (LAT) are further assessed with BMD measurement. Where probabilities calculated using BMD lie above or below the intervention threshold (IT), treatment or lifestyle advice, respectively, is recommended [3, 90]. Patients with probabilities above the upper assessment threshold (UAT) are considered for treatment. Those with probabilities above the very high-risk threshold (VHRT) should be considered for specialist referral. Where BMD measurement is not practical (e.g., when individuals are frail and unable to get onto a DXA table, or lie flat on a DXA table), patients with probabilities above the IT are considered for treatment

1. An initial FRAX assessment, which provides the ten-year probability of a major osteoporotic fracture (MOF; clinical spine, hip, forearm or humerus) and/or hip fracture, can be used to identify patients at low, intermediate, high or very high risk of fracture (Strong recommendation).
2. Consider, particularly in older people, drug treatment in those with a prior and/or recent fragility fracture, with fracture risk assessment informing the choice of drug treatment (Strong recommendation).
3. Men and women with *high and very high fracture risk* (*see *Fig. 1) should have a DXA if a baseline measurement is needed against which to compare future BMD measurements (Strong recommendation).
4. Men and women with *intermediate fracture risk* (i.e., between the upper and lower assessment thresholds) should be referred for BMD measurement, if practical. Thereafter, fracture probability should be reassessed using FRAX (Strong recommendation).
5. When BMD is included in a FRAX assessment, the patient’s risk (high, very high or low) is determined by the higher of the two (MOF and hip fracture) risk assessments (Strong recommendation).
6. In men and women with *intermediate fracture risk*, if BMD measurement is unavailable, contraindicated, or impractical (e.g., in frail individuals), drug treatment should be offered if there is a history of fragility fracture and/or if fracture risk exceeds the intervention threshold (Strong recommendation).
7. Men and women with *low fracture risk*, without a prior fragility fracture, can be reassured that their fracture risk is low and offered lifestyle advice as appropriate (Strong recommendation).
8. Consider referral of very high-risk patients to an osteoporosis specialist in secondary care for assessment and consideration of parenteral treatment (some may need first-line anabolic drug treatment, especially those with multiple vertebral fractures). Indications of very high risk, where specialist referral should be considered, include (Conditional recommendation):
  The presence of single but important clinical risk factors, such as
    A recent vertebral fracture (within the last 2 years)
    ≥ 2 vertebral fractures (whenever they have occurred)
    BMD T-score ≤  − 3.5
    Treatment with high-dose glucocorticoids (≥ 7.5 mg/day of prednisolone or equivalent over 3 months) (refer urgently given rapid loss in bone post initiation of glucocorticoids; if any delay is anticipated, start an oral bisphosphonate in the meantime)
  The presence of multiple clinical risk factors, particularly with a recent fragility fracture indicating high imminent risk of re-fracture,
  Or other indicators of very high fracture risk, including as defined by FRAX.
9. The choice of drug treatment should be informed by the level of fracture risk, additional clinical risk factors, cost-effectiveness of treatment, and patient preferences (Strong recommendation).
10. FRAX and the link to the NOGG website should be incorporated into electronic patient health record systems (Strong recommendation).

### FRAX assessment thresholds for 10-year probability of fracture

**Fig. 2:**
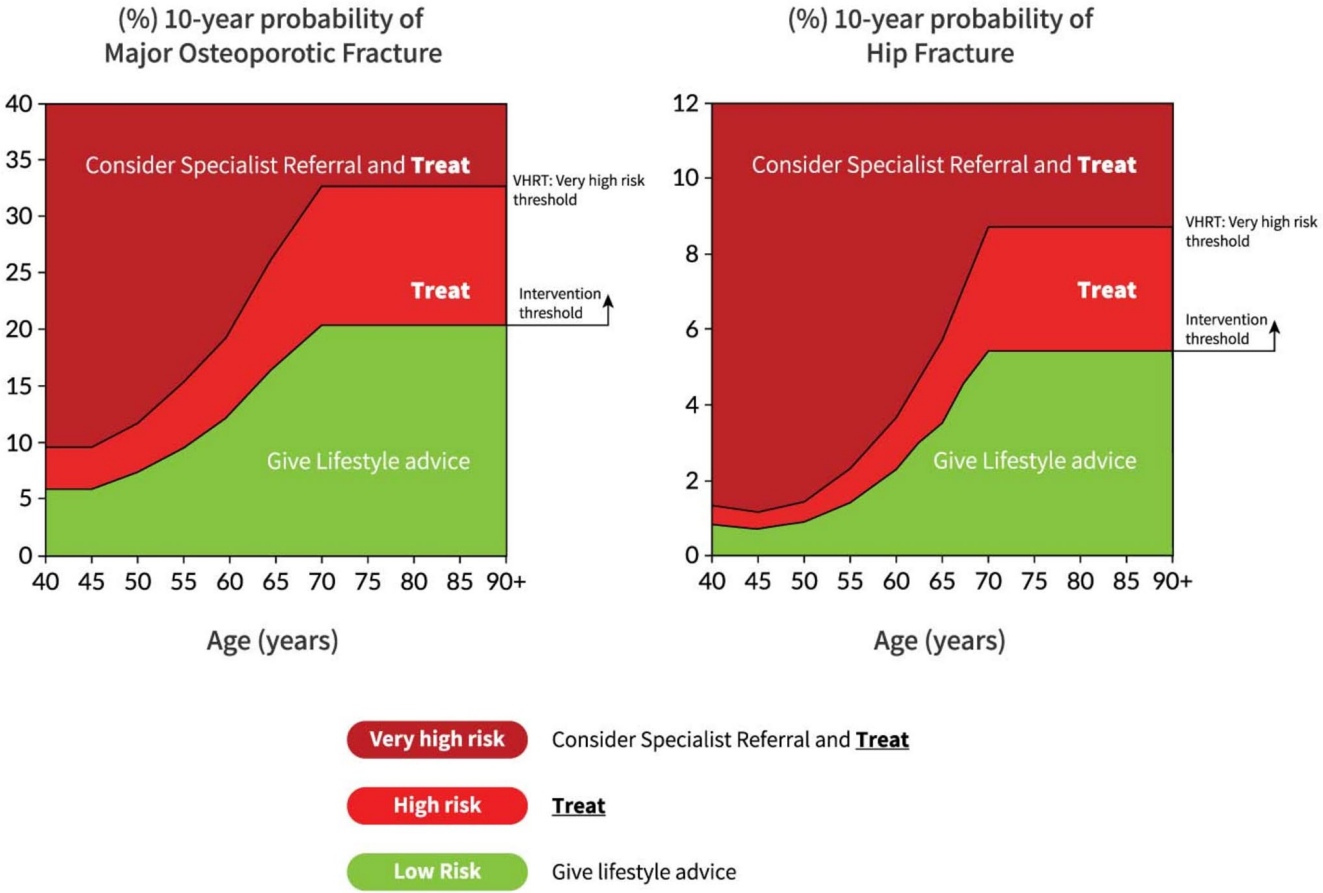
NOGG thresholds for intervention and/or referral using major osteoporotic fracture (MOF) and hip fracture (HF) probabilities in the UK. The panels show the thresholds following the recalculation of FRAX after the input of BMD; the same thresholds are used when BMD is unavailable. The intervention threshold (IT) and very high-risk threshold (VHRT) denote the thresholds for high and very high risk, respectively

The approach recommended for decision-making is based on fracture probabilities derived from FRAX and can be applied to men and women [90]. This approach is underpinned by cost-effectiveness analysis with oral or intravenous bisphosphonates as the intervention [116, 117]; (Evidence level Ib). FRAX assessment thresholds for 10-year probability of a major osteoporotic fracture (MOF) are shown in Fig. 1. An intervention threshold denotes a probability of fracture above which it is expected that a patient is offered anti-osteoporosis treatment. The fracture probability can be refined by DXA measurement of BMD and hence its relation to the intervention threshold. The use of FRAX without BMD has approximately the same performance as BMD without FRAX [11]; (Evidence level Ia). Thus, the same intervention threshold can be used when fracture risk is assessed with or without BMD (see Fig. 1). For men and women, the intervention threshold up to age 70 years is set at a risk equivalent to that of a woman of the same age with a prior fracture, in line with current clinical practice, and therefore rises with age. At age 70 years and above, fixed thresholds are applied [118]; (Evidence level Ib). The proportion of women potentially eligible for treatment rises from approximately 30 to 50% with age, largely driven by the prevalence of prior fracture [118]; (Evidence level Ib). When FRAX is calculated with BMD included, the NOGG website also provides intervention thresholds based on the 10-year probability of hip fracture, in addition to the 10-year probability of a MOF (Fig. 2). If there is discordance between the risk categories identified by the two probabilities, the highest risk category can be used to guide intervention. Of note, in the SCOOP study of screening for high fracture risk, treatment was targeted on the basis of risk assessed by hip fracture probability, with or without BMD [119].

### Indications for specialist referral in those at very high fracture risk

Individuals at very high fracture risk have the most to gain from thorough investigation of osteoporosis, falls assessment, and development and delivery of a personalised treatment plan for a chronic, life-long condition. A range of treatments now available to treat osteoporosis are mostly (but not exclusively) initiated through secondary care and the sequence in which they are used is important. Three anabolic agents (teriparatide, abaloparatide and romosozumab) are now available, with teriparatide licensed for a once-only treatment course. Within the licensing permissions, all anabolic agents might be used at different stages within the course of a lifetime of osteoporosis; however, there is currently no evidence to support a specific sequence, and input from an osteoporosis specialist is essential to inform a personalised care plan. Treatment with teriparatide or abaloparatide, which are anabolic skeletal agents, or romosozumab, which has a dual anabolic and antiresorptive action, results in rapid and greater fracture risk reductions than some antiresorptive treatments [120–122] [123]; (Evidence level Ib). This has led to the need to identify the sub-group of patients at very high fracture risk who would potentially benefit from clinical review by an osteoporosis specialist and who may benefit from anabolic drug treatment [124]. Indications for referral to an osteoporosis specialist may arise through several routes, for example, in the presence of single but important clinical risk factors, such as a recent vertebral fracture (within the last 2 years), ≥ 2 vertebral fractures (whenever they have occurred), a BMD *T*-score ≤ −3.5, high-dose glucocorticoids use (≥ 7.5 mg/day of prednisolone or equivalent over 3 months) [67, 125]; (Evidence levels IIb and IV), or via a combination of clinical risk factors, resulting in very high fracture risk [126]; (Evidence level IIb).

Prior fragility fracture is a well-established risk factor for a future fracture. This risk of subsequent osteoporotic fracture is particularly acute immediately after an index fracture and wanes progressively over the next 2 years, but thereafter remains higher than that of the general population [103, 127–134]. This effect of recency of fracture, sometimes termed imminent risk [133], is also dependent on age, sex, and site of fracture [39]; (Evidence level Ic). This complexity is being addressed by the development of optional post-FRAX algorithms to allow clinicians to explore the potential impact of fracture recency on the calculated probability of MOF and hip fracture (see Table 1) [39]. The mechanism underlying imminent risk is not yet fully understood and no clinical risk factors have yet been identified for short-term recurrent fractures that differ from those identified for fracture over a longer time horizon [89]. Few therapeutic studies have reported the recency of fracture in those patients whom they have recruited, though rapid clinical efficacy has been demonstrated within studies of zoledronate, risedronate, teriparatide, abaloparatide, and romosozumab [121, 135, 136] [123]; (Evidence level Ib).

**Fig. 3:**
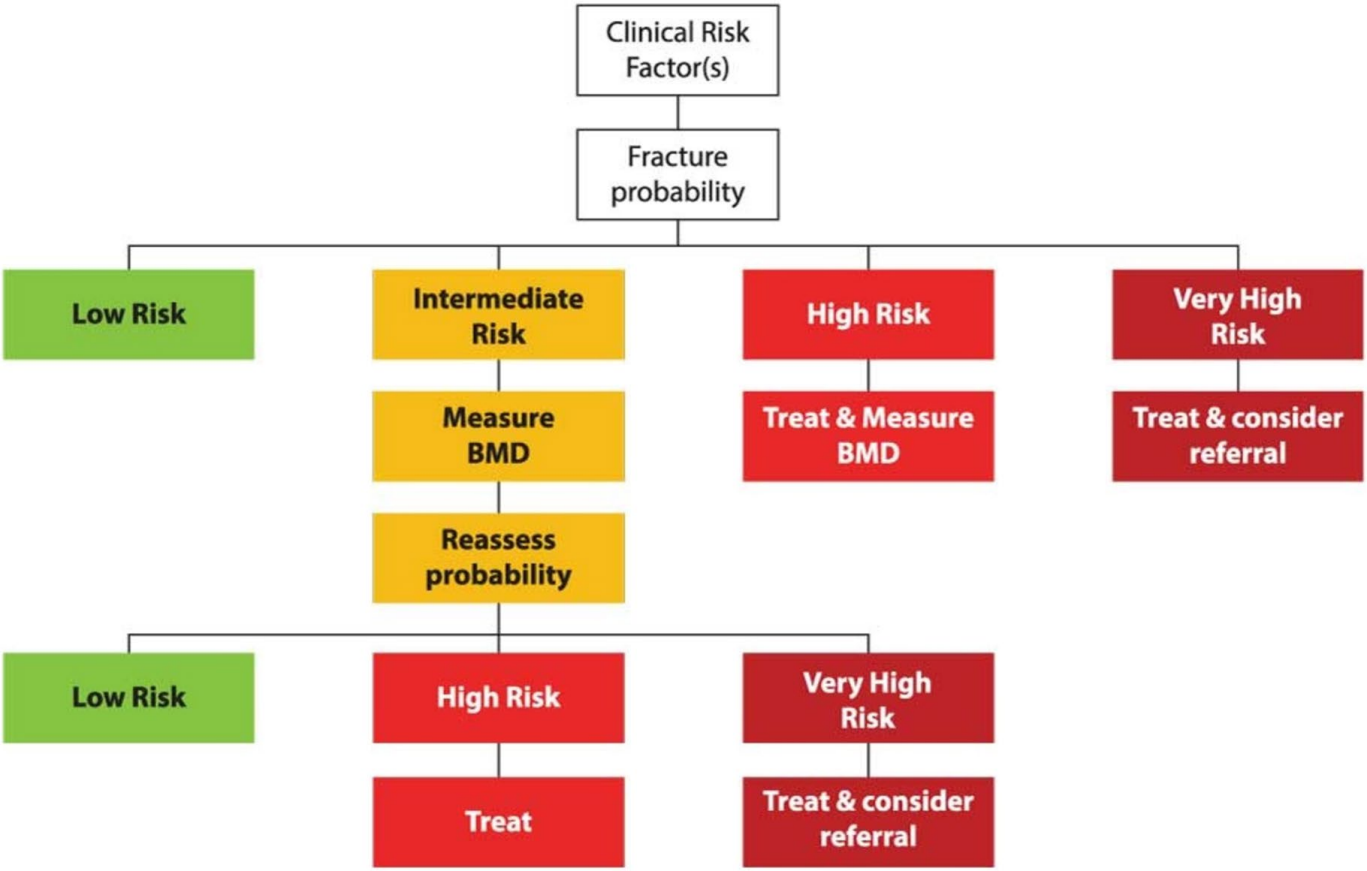
Management algorithm for the assessment of individuals at risk of fracture [137]. Those at very high risk should be treated and considered for referral to an osteoporosis specialist in secondary care; some may benefit from parenteral treatment (including first-line anabolic drug treatment, especially if multiple vertebral fractures). All individuals should be offered lifestyle advice. CRF, clinical risk factor

A NOGG threshold that characterises men and women at high and very high fracture risk has also been established using FRAX probabilities; very high risk is identified as a FRAX-based fracture probability that exceeds the intervention threshold by 60% (Figs. 1 and 2) [137]. It can be used to identify patients who likely require specialist referral for assessment of their osteoporosis (which should include DXA measurement of BMD), and further consideration of appropriate treatment strategies [124, 138]. The proportion of postmenopausal women at very high risk defined in this way rises from approximately 6% at age 50–54 to 36% at age 90 years or older. Numerical values for the probability thresholds are given in Table 4 for MOF and for hip fracture. An assessment algorithm is shown in Fig. 3. In patients with FRAX probabilities in the high-risk category, consideration of additional clinical risk factors (e.g., frequent falls, very low spine BMD; see Table 1) can also lead to redesignation from high to very high risk of fracture.

**Table 4:** Numerical values for NOGG thresholds for major osteoporotic fracture and hip fracture probabilities based on FRAX

Age (years)	LAT	IT	UAT	VHRT
**Major osteoporotic fracture**				
50	3.4	7.3	8.8	11.7
55	4.5	9.5	11.4	15.2
60	6.0	12.2	14.6	19.4
65	8.6	16.5	19.8	26.4
70	11.1	20.3	24.4	32.5
**Hip fracture**				
50	0.23	0.91	1.1	1.5
55	0.43	1.5	1.7	2.3
60	0.80	2.3	2.8	3.7
65	1.4	3.5	4.2	5.6
70	2.6	5.4	6.5	8.6

LAT and UAT refer to the lower and upper assessment thresholds, respectively, between which a BMD is indicated. The intervention threshold (IT) and very high-risk threshold (VHRT) denote the thresholds for high and very high risk

### FRAX—practical considerations

The FRAX MOF probabilities are transferred automatically to the NOGG website by clicking on the specified button on the FRAX results box. Where practitioners receive the results of a FRAX risk assessment for an individual patient without treatment guidance, the FRAX probabilities can also be entered manually onto the NOGG website (https://www.nogg.org.uk/manual-data-entry); this page also captures additional information (age, sex, glucocorticoid exposure and finally, whether a femoral neck BMD has been included, in the FRAX estimates) so that the result can be automatically compared to the NOGG thresholds with appropriate guidance on treatment. In the case of a patient born in another country, now living in the UK, FRAX-derived probabilities from a non-UK FRAX tool can be entered manually onto the NOGG website (htts://www.nogg.org.uk/manual-data-entry) to determine where risk lies in relation to intervention thresholds. Lack of integration of FRAX assessments and links to NOGG guidance in existing patient health record systems represents a barrier to effective fracture risk assessment (Evidence IV).

The targeted use of BMD assessments with the NOGG strategy makes more efficient use of often limited resources than would DXA scanning of all individuals with risk factors [139]; (Evidence level Ib). Historically it was thought that treatment should not be undertaken in women without initial BMD measurement, except in those with hip or vertebral fractures. This view arose after a post-hoc analysis in 1998 suggested reduced efficacy of alendronate in patients with BMD *T*-scores above − 2.5 [140]; (Evidence level Ib). However, this approach is now outdated as many studies have since shown little or no interaction of BMD on the effectiveness of several agents, including bisphosphonates (e.g., zoledronate, denosumab, raloxifene, abaloparatide and teriparatide) [75, 141–144] [145] [146]; (Evidence level Ib). Moreover, clinical risk factors are not totally independent of BMD and, when clinical risk factors alone are used in women age 70 years or more to identify patients at high fracture risk, BMD is approximately 1 SD lower in the high-risk group compared with a low-risk group [147, 148]; (Evidence level Ib). These findings indicate that the categorisation of patients at high fracture risk on the basis of FRAX without BMD mostly selects patients with low BMD and that the higher the fracture probability, the lower the BMD. Note that this does not preclude the use of DXA scanning if more widely available; in addition to providing the most accurate risk assessment, DXA provides a baseline measurement for treatment monitoring and also permits, again if available and indicated, detection of vertebral fractures using VFA. FRAX is not recommended as a tool to monitor treatment [149]; (Evidence level IIb). However, the use of FRAX is appropriate to re-evaluate current fracture probabilities when considering a change in patient management; (Evidence level IV).

## Non-pharmacological management of osteoporosis

### Recommendations

#### Postmenopausal women and men, age ≥ 50 years, with osteoporosis or who are at risk of fragility fracture are recommended:

1. A healthy, nutrient-rich balanced diet (Strong recommendation).
2. An adequate intake of calcium (minimum 700 mg daily) preferably achieved through dietary intake or otherwise by supplementation (Strong recommendation).
3. To consume vitamin D from foods or be prescribed vitamin D supplements of at least 800 IU/day if they have identified vitamin D insufficiency or risk factors for vitamin D insufficiency. Those who are either housebound or living in residential or nursing care are more likely to require calcium and vitamin D supplementation to achieve recommended levels of intake (Strong recommendation).
4. A combination of regular weight-bearing and muscle strengthening exercise, tailored according to the individual patient’s needs and ability (Strong recommendation).
5. Advice about smoking cessation if an individual is a smoker (Strong recommendation).
6. Advice to restrict alcohol intake to ≤ 2 units/day (Strong recommendation).
7. A falls assessment should be undertaken in all patients with osteoporosis and fragility fractures; those at risk should be offered exercise programmes to improve balance and/or that contain a combined exercise protocol (Strong recommendation).

#### Dietary modification

A meta-analysis of observational studies examining different dietary patterns found a modest reduction in the risk of low BMD and of hip fractures in subjects adhering to ‘healthy’ (high in fruit and vegetables, fish, poultry and whole grains) diets and a reduction in the risk of low BMD in those with ‘milk/dairy’ diets. By contrast, those with a ‘meat/Western’ dietary pattern (high in processed and red meat, animal fat, refined sugar and soft drinks) saw a modest increase in the risk of low BMD and of hip fractures. However, population heterogeneity with the inclusion of subjects aged under 25 years in many dietary studies reduces generalisability [150]; (Evidence level IIa). A randomised controlled trial of a ‘healthy diet’ consumed for 30 days, specifically a calcium-rich diet that emphasises fruits, vegetables and low-fat dairy products (Dietary Approaches to Stop Hypertension (DASH)), resulted in a reduction in bone turnover [151]; (Evidence level Ib).

Protein is an important constituent of bone and muscle tissue, and good dietary intake is necessary to maintain the health of the musculoskeletal system. Protein intakes higher than the recommended daily allowance (RDA) of 0.75 g/kg body weight/day are associated with higher BMD at the neck of femur and total hip in one RCT, and in observational studies, has been associated with a reduced risk of hip fractures [152, 153]; (Evidence levels Ib and IIa); however, in a meta-analysis of 30 interventional studies, no significant effects of protein supplementation on BMD were seen [153]; (Evidence level Ia). Post-operative protein supplementation in patients with a recent hip fracture has been shown to improve the subsequent clinical course by significantly lowering rates of infection and duration of hospital stay [154]; (Evidence level Ib).

Whilst there are inconsistencies in the evidence base for the associations between vegetarian and vegan diets and musculoskeletal health, consumption of a vegetarian or vegan diet has been associated with lower BMD at the lumbar spine and hip than an omnivore diet, and a vegan diet has been associated with higher fracture risk [155]; (Evidence level IIa). A subsequent prospective cohort study of 65,000 people in the UK also identified lower BMD at the spine and hip in vegans and vegetarians, and higher hip fracture risk in vegans, attenuated in part by adjustment for calcium and/or protein intake [156]; (Evidence level IIb).

#### Calcium and vitamin D

At every stage of life, adequate dietary intakes of key bone nutrients such as calcium and vitamin D contribute to bone health. The UK Reference Nutrient Intake per day of calcium is 700 mg for adults aged 19 years and older [157]. Dietary calcium calculators are available to assess intake, e.g., https://www.cgem.ed.ac.uk/research/rheumatological/calcium-calculator/. Whilst the Scientific Advisory Committee on Nutrition (SACN) recommends a reference nutrient intake (RNI) of 400 IU daily of vitamin D for adults of all ages [158], in the context of osteoporosis, higher levels—specifically 800 up to 2000 IU daily may—be appropriate [159]; (Evidence level IV).

Most randomised controlled trials of anti-resorptive and anabolic drugs have included co-administration of calcium and vitamin D supplements. There have been many randomised controlled trials of either calcium alone, vitamin D alone, or both in combinations to examine whether use of these supplements alone reduces fracture risk. With respect to combined calcium and vitamin D supplements, meta-analyses have reported a reduction in hip and non-vertebral fractures, and possibly also in vertebral fractures [160–162]; (Evidence level Ia). Overall, there is little evidence that vitamin D supplementation alone reduces fracture incidence, although it may reduce falls risk [162, 163]; (Evidence level Ib). However, it is important for patients taking anti-resorptive and anabolic osteoporosis drug therapies to be vitamin D replete. In clinical practice, dietary sources of calcium are the preferred option, and calcium (combined with vitamin D) supplementation should be targeted to those who do not get sufficient calcium from their diet and who are at risk of osteoporosis and/or fragility fracture, such as older adults who are housebound or living in residential or nursing care [161], and those with intestinal malabsorption, e.g., due to chronic inflammatory bowel disease or following bariatric surgery. Calcium and vitamin D supplements may increase the risk of kidney stones but not the incidence of cardiovascular disease or cancer [164]; (Evidence level Ia). Routine intermittent administration of large doses of vitamin D, e.g. ≥ 60,000 IU, is not advised, based on reports of an associated increased risk of fracture and falls [165, 166]; (Evidence level Ia).

#### Exercise to improve or maintain bone density

Exercise has beneficial effects on BMD [167] (Evidence level Ia); however, clear evidence for a reduction in fracture risk is wanted. The effect of exercise on different skeletal sites varies. Combination exercise programmes, which include weight-bearing and resistance strengthening exercise, are effective at reducing bone loss in the femoral neck and lumbar spine in post-menopausal women [167, 168]; (Evidence level Ia). Similarly, upper body resistance exercise increases forearm bone mass [169]; (Evidence level Ia). A meta-analysis of the effects of exercise interventions on BMD in men found only three studies and identified a significant but moderate improvement in BMD at the femoral neck and a trend towards increased BMD at the lumbar spine [170]; (Evidence level Ia).

The effect of exercise varies with intensity and duration. Strengthening (resistance) exercise may be more effective if supervised. People at risk of falls, or with vertebral fractures, may need more specific advice and assessment before increasing exercise intensity [171]. The NOGG supports the Royal Osteoporosis Society Strong, Steady and Straight Expert Consensus Statement, which offers advice on intensity and duration and linked patient information videos and factsheets [171]. In people with osteoporosis, repetitive forced spinal forward flexion exercises should be undertaken with care as this specific movement may be associated with an increased risk of new vertebral fractures [172]; (Evidence level Ia). However, in general, people with osteoporosis can safely participate in exercise because the risk of serious adverse events is very low [172]; (Evidence level Ia).

#### Falls interventions

The majority of non-vertebral fractures are preceded by a fall. Exercise can significantly reduce the risk of falls and, perhaps, the risk of subsequent fractures, by maintaining or restoring muscle strength, balance and posture, improving confidence and reaction times. However, two recent large randomised controlled trials have not demonstrated an effect of multi-disciplinary interventions, targeted at falls, on fracture reduction when combined with screening for falls risk in primary care [173, 174]; (Evidence level Ib), a recent Cochrane review of falls prevention exercise programmes, and two previous meta-analyses demonstrated, albeit with low certainty, evidence of a reduction in fall-related fractures (or falls resulting in fractures) in those living in the community [168, 175, 176]; (Evidence level Ia). Exercise interventions to reduce falls in people with osteoporosis and/or at high risk of falling have been found to be safe [177]; (Evidence level Ia). Programmes that involve balance training and/or a combined exercise protocol are more effective in those who have risk factors for falling [175, 177]; (Evidence level Ia). Combined exercise protocols may include resistance training, balance challenging, aerobic exercise and impact exercise. Interventions of 3 h per week or more are most effective [178]; (Evidence level Ia). Interventions of short duration (less than 6 months) have been found to be effective, and good compliance with exercise interventions has been reported [177]; (Evidence level Ia). Home safety interventions (best delivered by an occupational therapist) have been shown to reduce the risk of falls in people living in the community [179]; (Evidence level Ia). Furthermore, whole body vibration has been demonstrated to reduce fall rate but does not increase BMD [180]; (Evidence level Ia).

#### Lifestyle measures

Other measures to improve bone health include optimisation of body mass index if underweight or overweight, stopping smoking, and reducing alcohol intake. Smoking cessation has been demonstrated to reduce the risk of vertebral and hip fractures in women [181, 182]; (Evidence levels Ilb and IIa). However, the risk of hip fracture was reduced in those who had stopped smoking, compared with current smokers, only after 5 years. Furthermore, pre-operative smoking cessation is associated with fewer post-operative complications [183]; (Evidence level Ia). Currently, minimal evidence is available on the fracture risk associated with vaping/e-cigarette use; one cross-sectional study has identified elevated risk of self-reported fragility fracture in electronic cigarette users [184]; (Evidence level Ic). In men with previous alcohol dependence, BMD is significantly lower than controls, but improves following 3–4 years of abstinence [185]; (Evidence level IIa). National guidelines recommend alcohol intake is limited to ≤ 2 units/day for women and men [186].

## Pharmacological treatment options

### Recommendations

1. Fracture risk assessment, patient suitability and preference should inform the choice of drug treatment. In most people at risk of fragility fracture, anti-resorptive therapy is the first-line option (Strong recommendation).

#### Antiresorptive drug treatment

2. Offer oral bisphosphonates (alendronate or risedronate) or intravenous zoledronate as the most cost-effective interventions. Alternative options include denosumab, ibandronate, hormone replacement therapy, raloxifene, and strontium ranelate (Strong Recommendation).
3. Offer intravenous zoledronate as a first-line treatment option following a hip fracture (Strong Recommendation).
4. Consider offering younger postmenopausal women (age ≤ 60 years) with high fracture risk and low baseline risk for adverse malignant and thromboembolic events HRT as a first-line treatment option (Conditional recommendation).
5. Discuss continued use of HRT after the age of 60 years with the patient, with treatment based on an individual risk–benefit analysis (Conditional recommendation).
6. When HRT is discontinued, reassess fracture risk and consider an alternative treatment if indicated (Conditional recommendation).
7. Before starting denosumab, ensure that a long-term personalised osteoporosis management plan is in place and that both the patient and the primary care practitioner are made aware that denosumab treatment should not be stopped or delayed without discussion with a healthcare professional (Strong recommendation).
8. Avoid unplanned cessation of denosumab because it can lead to increased vertebral fracture risk; hence, it must not be stopped without considering an alternative therapy (Strong recommendation).
9. If denosumab therapy is stopped, intravenous infusion of zoledronate is recommended 6 months after the last injection of denosumab, with subsequent monitoring of serum CTX guiding the timing of further treatment (Strong Recommendation). Where monitoring of serum CTX is not possible, consider a further intravenous infusion of zoledronate 6 months after the first dose of zoledronate (Conditional Recommendation).

#### Anabolic drug treatment

10. Consider teriparatide, abaloparatide or romosozumab as first-line treatment options in postmenopausal women at very high fracture risk, particularly in those with vertebral fractures (Conditional Recommendation).
11. Consider teriparatide as a first-line treatment option in men aged 50 years and older who are at very high fracture risk, particularly in those with vertebral fractures (Conditional Recommendation).
12. Consider as second-line treatment options, teriparatide in postmenopausal women and men age 50 years and older, and abaloparatide or romosozumab in postmenopausal women who are intolerant of bisphosphonate treatment, particularly in those with vertebral fractures (Conditional recommendation).
13. Following the approved duration of treatment with teriparatide, abaloparatide, or romosozumab (24, 18 or 12 months respectively), initiate treatment with alendronate, zoledronate, or denosumab without delay (Strong Recommendation).
14. Consider raloxifene as an option for follow-on treatment after an anabolic drug in women (Conditional recommendation).

#### Other treatments

15. When other antiresorptive and anabolic treatments are contraindicated or not tolerated, strontium ranelate can be used to treat postmenopausal osteoporosis and men with severe osteoporosis, provided the risk–benefit in relation to cardiovascular and thromboembolic events is considered. Initiation by a specialist who is an expert in osteoporosis management is advised (Strong recommendation).
16. Offer calcium and/or vitamin D supplementation as an adjunct to anti-osteoporosis drug treatment, if dietary calcium is low and/or vitamin D insufficiency is a risk, respectively (Strong recommendation).
17. Treat vitamin D deficiency and insufficiency prior to initiation of parenteral anti-osteoporosis drug treatment, and alongside initiation of oral anti-osteoporosis drug treatment (Strong recommendation).

### Overview of treatment options

Drugs used in the management of osteoporosis can be considered under two broad headings based on their primary mode of action. Anti-resorptive drugs primarily inhibit osteoclastic bone resorption with later secondary effects on bone formation. Anabolic drugs primarily stimulate osteoblastic bone formation with variable effects on bone resorption to increase bone density and strength. Most drugs fit into one or other category, but romosozumab has a dual action, both stimulating bone formation and inhibiting bone resorption. Teriparatide and abaloparatide both promote bone remodelling, increasing bone formation and, in response, bone resorption. Anti-resorptive drugs are much less expensive than anabolic drugs. It is important to consider the long-term management strategy for each patient initiated on osteoporosis treatment, as the timing of use of certain drugs is important. For example, teriparatide can only be used once as a full course of treatment in a lifetime (abaloparatide and romosozumab do not have this restriction), whilst denosumab requires careful consideration before initiation given the difficulties in stopping treatment once it is started.

The drugs listed in Table 5 have been shown to reduce fragility fractures in postmenopausal women, and men where indicated, with osteoporosis [187] (Evidence levels Ia and Ib). The efficacy of the drugs listed in Table 5 is well established for the prevention of vertebral fractures. Teriparatide and romosozumab are superior to risedronate and alendronate respectively at reducing vertebral fractures in high-risk postmenopausal women with osteoporosis. Most drugs listed in Table 5 have been shown to reduce hip fracture incidence, with the exception of ibandronate, calcitriol, raloxifene, and abaloparatide. Drugs listed in Table 5 (except calcitriol and raloxifene) have been shown to reduce the incidence of non-vertebral fractures.

**Table 5:** Anti-fracture efficacy of approved drug treatments for postmenopausal women and men with osteoporosis when given with calcium and vitamin D

Intervention	Vs. placebo			Vs. another drug treatment			
	Vertebral fracture	Non-vertebral fracture	Hip fracture	Vertebral fracture	Non-vertebral fracture	Hip fracture	Licenced for use in men
Romosozumab	Ib	IIb	IIb	Superior to Alendronate (Ib)*	Superior to Alendronate (Ib)*	Superior to Alendronate (Ib)*	No
Teriparatide	Ia	Ia	Ia	Superior to Alendronate (Ia) Risedronate (Ia) Denosumab (Ia)	Superior to Alendronate (Ia)	NAE	Yes
Abaloparatide	Ia	Ia	IIb	Superior to Raloxifene (Ia)	Superior to Teriparatide (Ia)	NAE	No
Alendronate	Ia	Ia	Ia	Inferior to Teriparatide (Ia) & Romosozumab (Ib)	Inferior to Teriparatide & Abaloparatide (Ia)	Inferior to Romosozumab (Ib)	Yes
Ibandronate	Ib	Ib	NAE	NAE	NAE	NAE	No
Risedronate	Ia	Ia	Ia	Inferior to Teriparatide (Ia)	Inferior to Abaloparatide (Ia)	NAE	Yes
Zoledronate	Ia	Ia	Ia	NAE	NAE	NAE	Yes
Calcitriol	IIa	NAE	NAE	NAE	NAE	NAE	Yes
Denosumab	Ia	Ia	Ia	Inferior to Teriparatide (Ia)	NAE	NAE	Yes
HRT	Ia	Ia	Ia	NAE	NAE	NAE	No
Raloxifene	Ia	NAE	NAE	Inferior to Teriparatide & Abaloparatide (Ia)	NAE	NAE	No
Strontium Ranelate	Ia	Ia	IIb	NAE	NAE	NAE	Yes

Evidence levels are shown in Appendix 2

*HRT* hormone replacement therapy, *NAE* no available evidence from clinical trials

^*^When 12 months of Romosozumab is followed by an antiresorptive

#### Primary and secondary care drug initiation

Oral and intravenous bisphosphonates, denosumab, raloxifene, calcitriol, and HRT can be initiated by primary or secondary care clinicians. If denosumab is initiated in primary care, consultation with secondary care colleagues is advised given the need to have a long-term personalised osteoporosis management plan in place before denosumab is started to enable denosumab to be stopped in a managed way as necessary. As calcitriol use is only supported by a grade IIa evidence base, its use is generally restricted to a select sub-group managed through secondary care. Strontium ranelate can be initiated by primary or secondary care clinicians, but if started in primary care should involve consultation with secondary care.

#### Secondary care drug initiation

Teriparatide, abaloparatide and romosozumab should be initiated by secondary care clinicians. In the UK teriparatide and romosozumab are provided via ‘home healthcare’ services, which also provide patient education.

#### Considerations when choosing which anabolic agent to use

i. Teriparatide and romosozumab have been shown to reduce hip fracture risk, whereas this has not been demonstrated for abaloparatide in the setting of a randomised controlled trial.
ii. There are no consistent randomised controlled trials showing superiority or otherwise of abaloparatide against anti-resorptives for any fracture type. However, a systematic review and network meta-analysis of randomised controlled trials and real-world evidence has suggested that whilst abaloparatide is equivalent to teriparatide at reducing vertebral fracture risk, abaloparatide is superior to teriparatide at reducing non-vertebral fracture risk [188].
iii. There is no evidence for superiority or otherwise of abaloparatide or teriparatide against romosozumab.
iv. All three anabolic agents are approved for the treatment of osteoporosis in postmenopausal women at increased risk of fracture; however, only teriparatide is approved for use in men. NICE additionally recommends abaloparatide for people who identify as non-binary and trans men, registered female at birth, and romosozumab for people who are post-menopausal, not specifying sex.
v. Teriparatide is given for 24 months, abaloparatide for 18 months, and romosozumab for 12 months.
vi. Teriparatide and abaloparatide are injected once daily; romosozumab is injected once monthly.
vii. Teriparatide pens must be stored in the fridge. Unopened abaloparatide pens must be stored in the fridge before use; after opening they can be stored at room temperature for 30 days, after which they must be discarded.
viii. Unlike teriparatide and abaloparatide, which are single-effect anabolic agents, romosozumab has a dual action, conferring both anabolic and antiresorptive effects.

### Treatment sequence

Any patient stopping denosumab, romosozumab, teriparatide, or abaloparatide requires a sequential therapy strategy usually involving an anti-resorptive drug, which should be planned at the time the initial anabolic therapy is instigated to avoid a gap in treatment.

## Specific drug options

### Anti-resorptive drugs: bisphosphonates

Alendronate 70 mg once weekly by mouth is recommended for the treatment of women with postmenopausal osteoporosis (PMO), men with osteoporosis, glucocorticoid-induced osteoporosis (GIO) and the prevention of PMO and GIO. The 70 mg weekly dose is considered equivalent to the previously approved dose of 10 mg daily. In postmenopausal women with osteoporosis, alendronate has been shown to reduce vertebral, non-vertebral, and hip fractures [189]; (Evidence level Ib). Approval for the use of alendronate in men with osteoporosis, and in men and women taking glucocorticoids, was granted on the basis of BMD bridging studies [190, 191]; (Evidence level Ib). Although the daily dose of alendronate (10 mg) is licensed for use in men, this is considered equivalent to the weekly dose (70 mg) which is commonly prescribed off-licence; (Evidence level IV).

Common side-effects of alendronate include upper gastrointestinal symptoms, bowel disturbance, headaches, and musculoskeletal pain. Alendronate should be taken after an overnight fast and at least 30 min before the first food or drink (other than water) of the day or any other oral medicinal products or supplementation (including calcium). Tablets should be swallowed whole with a glass of plain water (~ 200 ml) while the patient is sitting or standing in an upright position. Patients should not lie down for 30 min after taking the tablet. Alendronate is also available as 70 mg effervescent or soluble tablets, to be dissolved in a glass of plain water (≥ 120 ml).

Risedronate 35 mg once weekly by mouth is recommended for the treatment of PMO, men with osteoporosis, GIO, and the prevention of GIO in women. The 35 mg weekly dose is considered equivalent to the previously approved dose of 5 mg daily. In postmenopausal women with osteoporosis, risedronate has been shown to reduce vertebral and non-vertebral fractures [192, 193]; (Evidence level Ib). In a large population of older women, risedronate significantly decreased the risk of hip fractures, an effect that was greater in osteoporotic women [76]; (Evidence level Ib). Approval for use of risedronate in men with osteoporosis and in postmenopausal women taking glucocorticoids was granted on the basis of BMD bridging studies [194–196]; (Evidence levels Ib).

Common side-effects include upper gastrointestinal symptoms, bowel disturbance, headache and musculoskeletal pain. Risedronate should be taken after an overnight fast and at least 30 min before the first food or drink (other than water) of the day or any other oral medicinal products or supplementation (including calcium). Tablets should be swallowed whole with a glass of plain water (≥ 120 ml) while the patient is sitting or standing in an upright position. Patients should not lie down for 30 min after taking the tablet.

Ibandronate 150 mg once monthly by mouth or 3 mg as a prefilled intravenous injection (usually given as a 15- to 30-s push via butterfly cannula) every 3 months is recommended for the treatment of postmenopausal women with osteoporosis. The 150 mg monthly dose and 3 mg 3-monthly intravenous dose are considered equivalent to 2.5 mg daily by mouth for the treatment of PMO. In postmenopausal women with osteoporosis, ibandronate 2.5 mg daily has been shown to reduce vertebral fracture incidence [197]; (Evidence level Ib). In a post-hoc analysis of women at high fracture risk (with a femoral neck BMD *T*-score below − 3.0), a significant reduction in non-vertebral fractures was shown [198]; (Evidence level Ib). No data are available to show efficacy of hip fracture risk reduction. Approval for the oral 150 mg once monthly and 3 mg intravenously every 3 months formulations was granted on the basis of BMD bridging studies [199, 200]; (Evidence levels Ib).

Common side-effects with the oral preparation include upper gastrointestinal side-effects and bowel disturbance. Intravenous administration may be associated with an acute phase reaction, characterised by an influenza-like illness; this is generally short-lived and typically occurs only after the first injection. Oral ibandronate should be taken after an overnight fast and 1 h before the first food or drink (other than water) of the day, or any other oral medicinal products or supplementation (including calcium). Tablets should be swallowed whole with a glass of plain water (180 to 240 ml) while the patient is sitting or standing in an upright position. Patients should not lie down for 1 h after taking the tablet.

Zoledronate 5 mg once yearly by intravenous infusion (as 5 mg/100 ml infusion given over a minimum of 15 min via an intravenous cannula) is recommended for the treatment of PMO, men with osteoporosis, and men and postmenopausal women with GIO. In postmenopausal women with osteoporosis, zoledronate 5 mg once yearly has been shown to reduce the incidence of vertebral, non-vertebral, and hip fractures [201]; (Evidence level Ib). Approval for use of zoledronate in men with osteoporosis and in men and women taking glucocorticoids was granted on the basis of BMD bridging studies [202, 203]; (Evidence levels Ib). When given shortly after hip fracture, men and women given zoledronate 5 mg annually had fewer clinical fractures and lower mortality 3 years later [136]; (Evidence level Ib). When given (without calcium supplementation) every 18 months to women with osteopenia, there were fewer vertebral and non-vertebral fractures [144, 204]; (Evidence level Ib). A lower, although non-significant, decrease in mortality in fracture-free women, fewer breast cancers, and fewer non-breast cancers were also reported as secondary outcomes by the end of the 6-year study.

Common side-effects include an acute phase reaction usually only after the first infusion [205], which can be ameliorated by co-administration of paracetamol. Glomerular filtration rate (eGFR) should be calculated prior to initiation of treatment and caution advised for recipients at risk of kidney failure, with monitoring for any increase in serum creatinine or reduction in eGFR. The MHRA recommends use of creatinine clearance instead of eGFR to inform treatment decisions in those aged over 75 years and/or with BMI < 18 or > 40 kg/m^2^. An increase in symptomatic atrial fibrillation, reported as a serious adverse event, was seen in the main phase III trial [201]; (Evidence level Ib).

### Contraindications and special precautions for the use of bisphosphonates

Oral and intravenous bisphosphonates are contraindicated in patients with hypocalcaemia, hypersensitivity to bisphosphonates, and in women who are pregnant or lactating. Oral bisphosphonates are contraindicated in people with abnormalities of the oesophagus that delay oesophageal emptying, such as stricture or achalasia, and inability to stand or sit upright for at least 30–60 min. They should be used with caution in patients with other upper gastrointestinal disorders. Zoledronate and risedronate are contraindicated in severe renal impairment (GFR ≤ 35 ml/min for zoledronate and ≤ 30 ml/min for risedronate), whilst alendronate and ibandronate are cautioned against (GFR ≤ 35 ml/min for alendronate and ≤ 30 ml/min for ibandronate). Pre-existing hypocalcaemia must be investigated and, where due to vitamin D deficiency, treated with vitamin D (e.g., 100,000 to 300,000 IU orally as a loading dose in divided doses) before zoledronate treatment is initiated.

### Anti-resorptive drugs: denosumab

Denosumab is a fully humanised monoclonal antibody against the Receptor Activator of Nuclear factor Kappa B Ligand (RANKL), a major regulator of osteoclast development and activity. It is approved for the treatment of PMO and men at increased fracture risk, for the treatment of bone loss associated with hormone ablation in men with prostate cancer at increased fracture risk, and for the treatment of bone loss associated with long-term systemic glucocorticoid therapy in adults at risk of fragility fracture [206]; (Evidence level Ib). Denosumab is given as a subcutaneous injection of 60 mg once every 6 months. It has been shown to reduce the incidence of vertebral, non-vertebral, and hip fractures in postmenopausal women with osteoporosis [207] and safety and efficacy are maintained over 10 years of treatment [208]; (Evidence level Ib). Approval for its use in men with osteoporosis was granted on the basis of a BMD bridging study [209]; (Evidence level Ib).

Denosumab is contraindicated in patients with hypocalcaemia or with hypersensitivity to any of the constituents of the formulation. Its use is not recommended in pregnancy or in those aged < 18 years. Hypocalcaemia, as a side-effect of denosumab treatment, increases with the degree of renal impairment; patients should be advised to report symptoms of hypocalcaemia. Pre-existing hypocalcaemia must be investigated and, where due to vitamin D deficiency, treated with vitamin D (e.g., 100,000 to 300,000 IU orally as a loading dose in divided doses) before denosumab treatment is initiated. Adequate intake of calcium and vitamin D is important in all patients, especially those with severe renal impairment. The SPC states all patients should have calcium checked prior to each dose. In patients predisposed to hypocalcaemia (e.g. patients with a creatinine clearance < 35 ml/min), serum calcium levels should also be checked within 2 weeks after the initial dose [210]. Side-effects include skin infection, predominantly cellulitis, eczema, hypocalcaemia, and flatulence.

Denosumab cessation leads to rapid reductions in BMD and elevations in bone turnover to levels above those seen before treatment initiation [211–213]; (Evidence level Ib). Patients who discontinue denosumab have an increased risk of sustaining multiple vertebral fractures. In a post hoc analysis of the FREEDOM study and its extension, women discontinuing denosumab had an increased rate of vertebral fracture over an average of 3–6 months since the last denosumab injection was due. Of those patients who sustained vertebral fractures, 60.7% sustained multiple fractures compared to 38.7% of those discontinuing placebo [214, 215]; (Evidence level Ib). The increase in vertebral fracture risk following cessation of denosumab therapy emphasises the need to continue treatment with an alternative anti-resorptive drug following denosumab withdrawal. An intravenous infusion of 5 mg of zoledronate, 6 months after the last denosumab injection, reduces subsequent bone loss [216–220], although this effect is not seen in all patients and may not be maintained beyond 1 year, particularly in those who have had more than 3 years of denosumab treatment [221] (Evidence levels IIa and IIb). Monitoring bone turnover markers at 3 and 6 months post zoledronate infusion can help guide the timing of subsequent infusions. Where bone turnover markers are not available, a second infusion of zoledronate after 6 months has been proposed [222]; (Evidence level IV). Oral alendronate 70 mg once weekly was shown to maintain BMD for 12 months in most patients following 1 year of denosumab therapy, although significant bone loss occurred in a minority [223]; (Evidence level IIa). Given the difficulties in stopping denosumab treatment, particularly careful consideration is needed before starting denosumab in younger postmenopausal women and men.

### Anti-resorptive drugs: hormone replacement therapy (HRT)

HRT comprises a large number of oestrogen or combined oestrogen plus progestogen formulations (including synthetic progestins or body identical progesterone, some of which are approved for the prevention of osteoporosis in postmenopausal women at risk of fragility fracture. Conjugated equine oestrogens 0.625 mg daily ± 2.5 mg/day of medroxyprogesterone acetate has been shown to reduce vertebral, non-vertebral and hip fracture risk in postmenopausal women not selected on the basis of low bone density or high fracture risk [224, 225]; (Evidence level Ib). The 2019 evidence review from NICE identified that HRT was associated with lower fracture risk or increased BMD in nine out of 10 analyses, summarised from two cohort studies, one RCT, a Cochrane review and one other systematic review [226]. The risk of venous thromboembolism (VTE) is increased by oral oestrogen containing HRT compared with baseline population risk, but not with transdermal HRT (at standard doses) [226]. HRT does not increase cardiovascular disease risk when started in women aged under 60 years [226]; when started within 10 years of menopause it appears to lower risk [227].

Some HRT preparations, particularly those containing synthetic progestins, are associated with a small excess incidence of breast cancer [228]. However, this risk appears less than that associated with adverse lifestyle factors such as reduced physical activity, obesity and high alcohol intake [229]. In women with a low underlying risk of breast cancer (i.e., most women), the benefits of HRT for up to 5 years’, when used for symptom relief alone without consideration of the added bone benefits, exceed potential harm [229].

### Anti-resorptive drugs: calcitriol

Calcitriol (1,25-dihydroxyvitamin D_3_) is the active form of vitamin D and, although rarely used now, is approved for the treatment of established postmenopausal osteoporosis in an oral dose of 0.25 µg twice daily. It acts mainly by inhibiting bone resorption. It has been shown to reduce vertebral fracture risk in postmenopausal women with osteoporosis, but effects on non-vertebral and hip fractures have not been demonstrated [230]; (Evidence level IIb). It is contraindicated in patients with hypercalcaemia or with metastatic calcification. Because calcitriol can cause hypercalcaemia and/or hypercalciuria, serum calcium and creatinine levels should be monitored at 1, 3, and 6 months after starting treatment and at 6-monthly intervals thereafter.

### Anti-resorptive drugs: raloxifene

Raloxifene is a selective oestrogen receptor modulator and inhibits bone resorption. It is approved for the treatment and prevention of osteoporosis in postmenopausal women. Raloxifene has been shown to reduce vertebral fracture risk, but reduction in non-vertebral and hip fractures has not been demonstrated [231]; (Evidence level Ib). Raloxifene is taken orally as a single daily 60 mg dose and may be taken at any time without regard to meals. Raloxifene is contraindicated in women with child-bearing potential, unexplained uterine bleeding, severe hepatic or renal impairment, and in women with a history of venous thromboembolism. Side effects include leg cramps, oedema, and vasomotor symptoms. There is a small increase in the risk of venous thromboembolism, mostly within the first few months of treatment, and a small increase in the risk of fatal stroke has been reported [232], (Evidence level IIa) such that it should be used with caution in women with a history of stroke or with risk factors for stroke disease. In the phase III trials, women treated with raloxifene had a significantly decreased risk of developing breast cancer [233]; (Evidence level Ib).

### Other drugs: strontium ranelate

Strontium ranelate is taken in a dose of 2 g once at night by mouth as a suspension of granules stirred in water, at least 2 h after food and/or consumption of calcium-containing products. As an alkaline earth metal (closely related to calcium) it substitutes for calcium within hydroxyapatite. Its mode of action is not completely understood, but the evidence suggests it has weak anti-resorptive effects whilst maintaining bone formation. In postmenopausal women with osteoporosis, strontium ranelate 2 g daily has been shown to reduce the incidence of vertebral and non-vertebral fractures [234, 235]; (Evidence levels Ib). Fewer hip fractures were reported in a post-hoc analysis of women at high risk of hip fracture (i.e., age ≥ 74 years with a femoral neck BMD *T*-score ≤  − 2.5). Approval for its use in men with osteoporosis was granted on the basis of a BMD bridging study [236]; (Evidence level Ib).

Common side effects include nausea and diarrhoea. There was a significant increase in venous thromboembolism in the Phase III trials [237]. Contraindications include previous myocardial infarction, stroke, or venous thromboembolism, as a post-hoc pooled safety analysis showed significant increases in myocardial infarction and ‘nervous system disorders ‘ including cerebrovascular disease, which was observed in patients taking strontium ranelate compared to placebo [238]. The manufacturer advises against use when the eGFR is < 30 ml/ml. The higher atomic number of strontium compared with calcium artefactually increases BMD when incorporated into the bone matrix [239]. When strontium ranelate is stopped, this effect is slow to resolve, with implications for future BMD monitoring.

### Anabolic drugs: teriparatide (recombinant human parathyroid hormone (PTH) 1–34)

When administered intermittently, teriparatide has anabolic skeletal effects which are most marked in trabecular bone. Teriparatide is approved for the treatment of osteoporosis in postmenopausal women and in men at risk of fragility fracture, and osteoporosis associated with systemic glucocorticoid therapy in women and men at risk of fragility fracture. Teriparatide is given as a subcutaneous injection in a dose of 20 µg/day. The duration of treatment is limited to 24 months.

Teriparatide is contraindicated in patients with hypercalcaemia, severe renal impairment, malignant disease affecting the skeleton, prior radiation to the skeleton, in women who are pregnant or lactating, and in metabolic bone diseases other than osteoporosis and osteogenesis imperfecta. Teriparatide should be used with caution in patients with moderate renal impairment. PTH levels need to be normal to initiate teriparatide; hence, levels should be checked even with normocalcaemia. Side effects include headache, nausea, dizziness, postural hypotension, and leg pain. Slight and transient elevations of serum calcium may occur following teriparatide injection.

Teriparatide has been shown to reduce vertebral and non-vertebral fractures in postmenopausal women with osteoporosis [240]; (Evidence level Ib). No primary efficacy end-point data are available for hip fracture incidence, but systematic review and meta-analysis level evidence has shown an OR for hip fracture risk of 0.44 (95% CI: 0.22, 0.87; *p* = 0.019) in patients treated with teriparatide compared with placebo, when considering hip fracture as a safety end point. No significant benefit was seen on upper limb fractures [241]; (Evidence level Ia). These findings were further supported by a network meta-analysis of a similar list of RCTs, which reported a HR of 0.35 (95% CI: 0.15, 0.73) for hip fracture in patients treated with teriparatide compared with placebo [242]; (Evidence level Ia). Approval for teriparatide use in men with osteoporosis and in men and women with glucocorticoid-induced osteoporosis was granted on the basis of BMD bridging studies [243, 244]; (Evidence level Ib). Teriparatide biosimilars are available which may improve the cost-effectiveness of teriparatide.

### Anabolic drugs: abaloparatide

Abaloparatide is a synthetic peptide analogue of the first 34 amino acids of the human parathyroid hormone-related peptide (PTHrP). It has anabolic skeletal effects and is approved for the treatment of osteoporosis in postmenopausal women at increased risk of fracture. Abaloparatide is given as a subcutaneous injection of 80 µg once daily. The duration of treatment is limited to 18 months. Abaloparatide is contraindicated in people with hypersensitivity to the active substance or to any of the excipients, pregnancy and breast-feeding, women of childbearing potential, pre-existing hypercalcaemia, severe renal impairment, unexplained elevations of serum alkaline phosphatase, patients with known risks for osteosarcoma such as those who have received prior external beam or implant radiation therapy involving the skeleton, and patients with skeletal malignancies or bone metastases. The European Medicines Agency SmPC specifies that patients should receive supplemental calcium and vitamin D if dietary intake is inadequate, and that no dosage adjustment is required in renal impairment; however, abaloparatide must not be used in people with severe renal impairment including patients with end-stage renal disease. Blood pressure, cardiac status and ECG should be assessed prior to beginning treatment with abaloparatide. Patients with cardiac disease should be monitored for worsening of their disease. Side-effects include hypercalcaemia and hypercalciuria, dizziness, back pain, nausea, headache, arthralgia, hypertension, injection site reaction, and palpitations.

Abaloparatide has been shown to reduce vertebral fractures in postmenopausal women with osteoporosis. There was no statistically significant reduction in non-vertebral fractures [123]; (Evidence level 1b). Two network meta-analyses have shown a reduction in both vertebral and non-vertebral fractures when compared to placebo, with RRs for vertebral fracture of 0.14 (95% CI 0.05, 0.42) [245] and 0.13 (95% credible interval (CrI) 0.04, 0.34) [246], and RRs for non-vertebral fracture of 0.51 (95%CI 0.29, 0.87) [245] and 0.50 (95% CrI 0.28, 0.85) [246]; (Evidence level Ia). A significant reduction in wrist fracture compared to placebo was also shown in one network meta-analysis (RR 0.39; 95% CrI 0.15, 0.90) [246] (Evidence level Ia). A further network meta-analysis of nine randomized controlled trials and two real-world evidence observational studies showed abaloparatide to be superior to teriparatide for non-vertebral fracture risk reduction (OR, 0.87; 95% CI, 0.80–0.95) [188]; (Evidence level Ia). There are insufficient trial data to assess efficacy against hip fracture.

### Anabolic drugs with additional antiresorptive effect: romosozumab

Romosozumab is a humanised monoclonal antibody that binds to and inhibits sclerostin. It has a dual action, stimulating bone formation and inhibiting bone resorption and is approved for the treatment of severe osteoporosis in postmenopausal women at very high risk of fracture. It is currently not approved for use in men, although it has been used successfully to increase BMD in men [247]; (Evidence level Ib). It is given as a subcutaneous injection in a dose of 210 mg (administered as two subcutaneous injections of 105 mg each) once monthly. The duration of treatment is limited to 12 months.

In postmenopausal women with osteoporosis who received romosozumab 210 mg or placebo subcutaneously once monthly for 12 months, followed by denosumab 60 mg subcutaneously in both groups for 12 months, new vertebral fractures and clinical fractures were significantly reduced in women treated with romosozumab when compared to placebo at 12 months, and at 24 months vertebral fracture rates were significantly lower in women treated with romosozumab during the first 12 months [120]; (Evidence Level Ib). In a comparator-controlled study in postmenopausal women with severe osteoporosis, subcutaneous romosozumab 210 mg once monthly for 12 months followed by oral alendronate 70 mg once weekly for 12 months was compared against alendronate 70 mg once weekly for 24 months [121]. New vertebral, non-vertebral, clinical, and hip fractures were all significantly lower in women treated with romosozumab followed by alendronate than in those treated with alendronate alone (Evidence level Ib). Significantly greater risk reduction in new vertebral and clinical fractures was seen for romosozumab vs alendronate at 12 months. A significantly higher incidence of cardiovascular events was seen in the romosozumab group compared to the alendronate group [120]; (Evidence level Ib).

Romosozumab is contraindicated in patients with hypocalcaemia, hypersensitivity to any of the constituents of the formulation, or a history of myocardial infarction or stroke. When determining whether to use romosozumab for an individual patient, both fracture and cardiovascular risk (based on risk factors) over the next year need to be considered. Transient hypocalcaemia has been observed in patients receiving romosozumab. Hypocalcaemia should be corrected prior to initiation of treatment, and patients should be adequately supplemented with calcium and vitamin D. Patients with severe renal impairment or on dialysis are at increased risk of developing hypocalcaemia. Osteonecrosis of the jaw and atypical femoral fractures have been very rarely reported with romosozumab use.

## Drug treatment for patients with very high fracture risk

Two randomised comparator-controlled studies in postmenopausal women with severe osteoporosis have demonstrated superior anti-fracture efficacy of skeletal anabolic agents versus anti-resorptive drugs. Subcutaneous romosozumab 210 mg once monthly resulted in significantly greater reduction of vertebral, non-vertebral, clinical, and hip fractures at 24 months (risk reduction of 48%, 19%, 27% and 38% respectively) and significantly greater risk reduction in new vertebral and clinical fractures at 12 months when compared to oral alendronate 70 mg once weekly. In the VERtebral fracture treatment comparisons in Osteoporotic women (VERO) study, subcutaneous teriparatide, 20 µg once daily, was associated with significantly fewer new vertebral and clinical fractures than oral risedronate, 35 mg once weekly (56% and 52% respectively) after 2 years of treatment [248]; (Evidence level Ib). These studies provide the rationale for considering teriparatide or romosozumab as a first-line treatment option in postmenopausal women at very high risk of fracture. Comparator studies of anti-resorptive and anabolic agents have not been reported in men.

Following discontinuation of treatment with teriparatide or romosozumab, bone turnover increases and there is a fall in BMD. Although not specifically studied for abaloparatide, similar changes would be expected. Since the maximum permitted duration of treatment with teriparatide is 24 months, with abaloparatide 18 months, and with romosozumab 12 months, sequential therapy with anti-resorptive drugs is required to maintain their beneficial skeletal effects. Both alendronate and denosumab have been shown to maintain and increase BMD at the spine and hip following teriparatide or romosozumab therapy [121, 249–252]. In the FRAME extension study, the beneficial effects of 12 months romosozumab therapy on vertebral and non-vertebral fracture risk were maintained when followed by 24 months of denosumab treatment [253]; (Evidence level IIb). Sequential therapy with alendronate following 18 months treatment with abaloparatide results in further gains in BMD and sustained reductions in vertebral and non-vertebral fracture rates [254]; (Evidence level IIb).

When women are switched from oral bisphosphonates to teriparatide or romosozumab, there is attenuation of the increase in spine and hip BMD compared to when these agents are used in treatment-naïve individuals. This blunting effect is greater for teriparatide than romosozumab, especially at the hip [255, 256]; (Evidence level IIb). The impact of these effects, if any, on fracture risk is unknown. The effect of switching from oral bisphosphonates to abaloparatide on the efficacy or effectiveness of abaloparatide treatment is unknown. In women previously treated with denosumab, switching to teriparatide is associated with transient bone loss in the spine and greater and longer lasting bone loss in the hip [250]. When romosozumab is given following denosumab therapy, there is attenuation of the BMD increase at the spine and hip [248, 257]; (Evidence level IIb). The impact of these effects, if any, on fracture risk is unknown.

## Duration and monitoring of bisphosphonate treatment

Osteoporosis is a long-term condition for which there is currently no cure; therefore, life-long treatment and monitoring to prevent fractures is often required.

### Recommendations

**Fig. 4:**
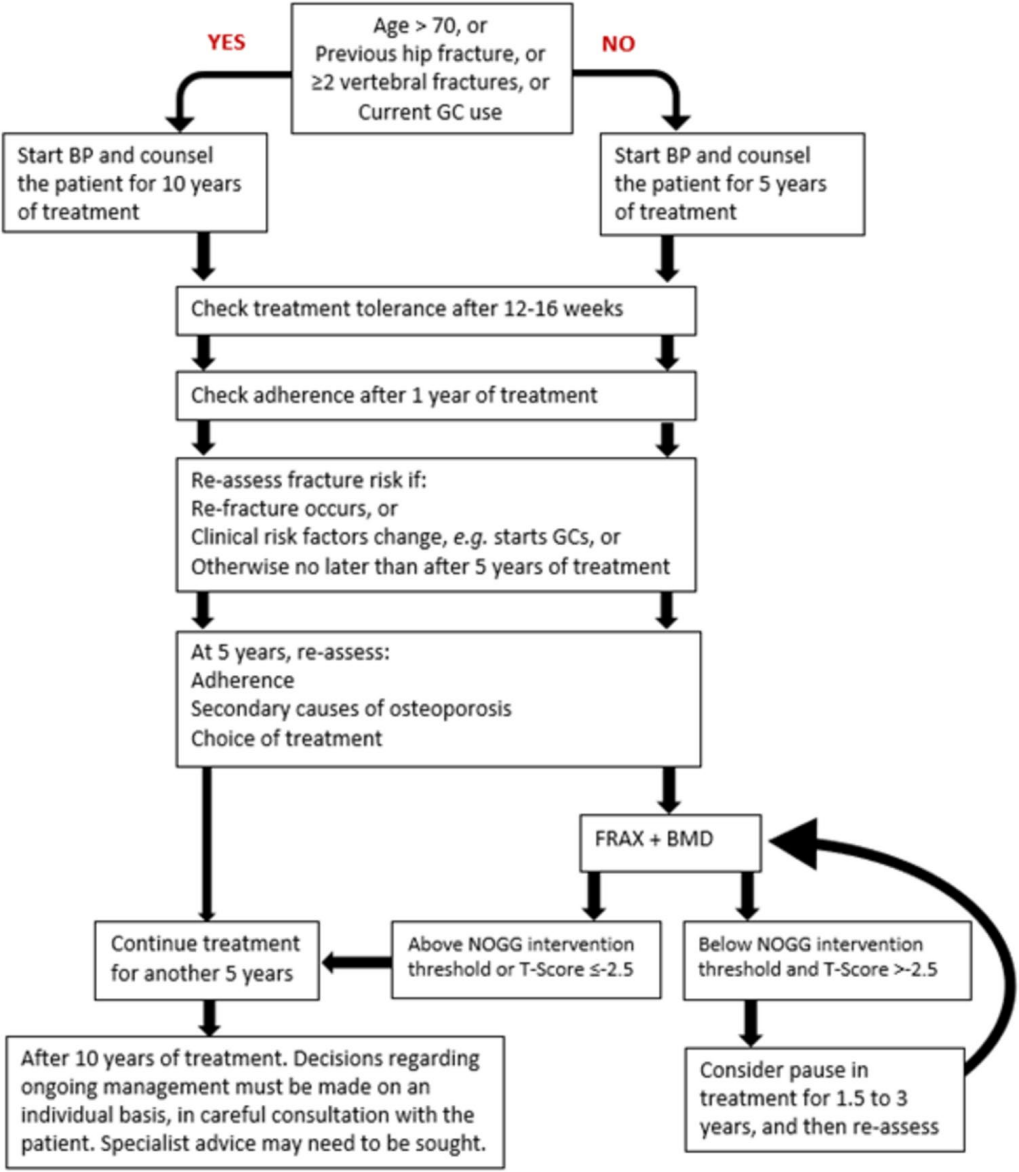
Oral bisphosphonates: clinical flowchart for long-term treatment and monitoring. GC, glucocorticoids (oral ≥ 7.5 mg prednisolone/day or equivalent); BP, bisphosphonate

**Fig. 5:**
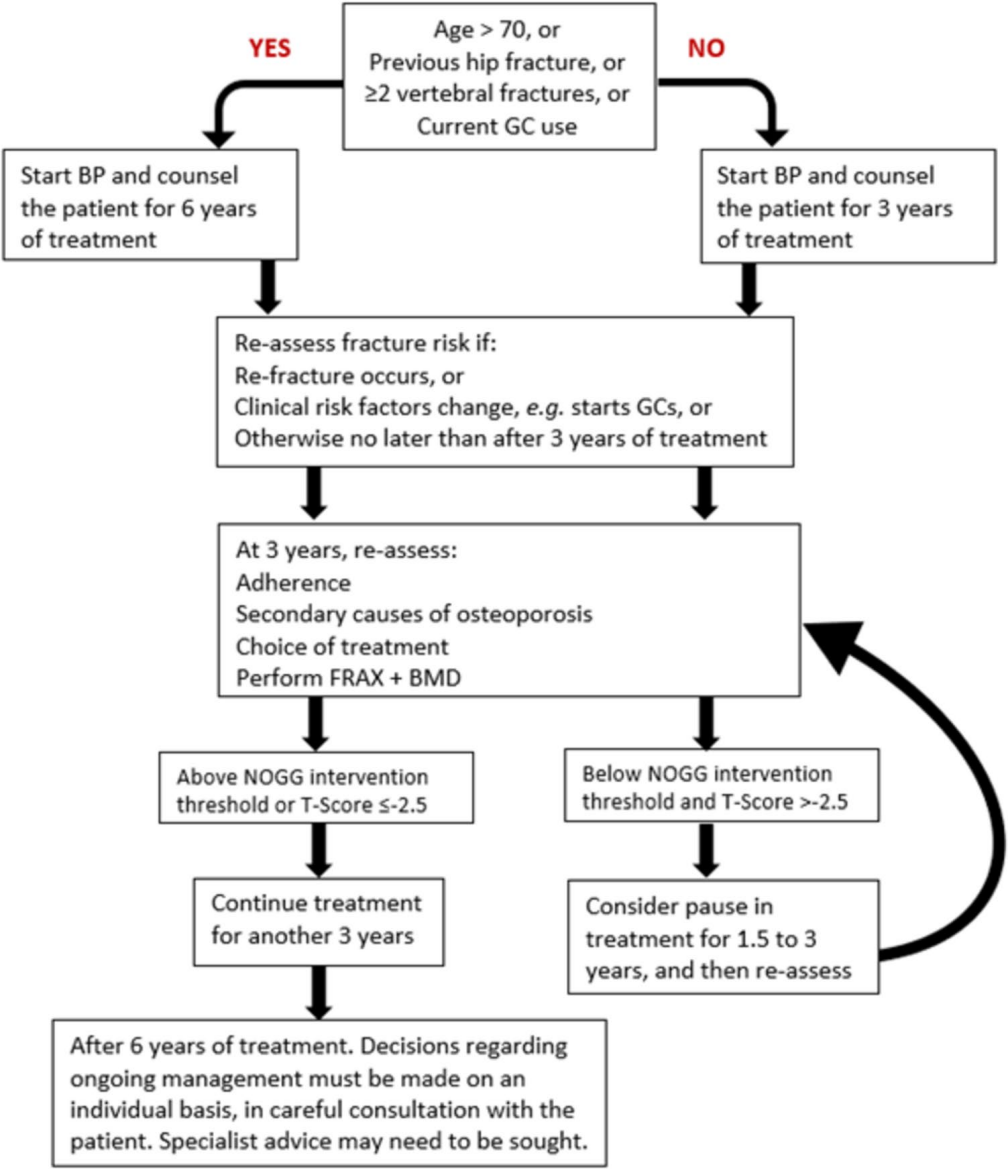
Intravenous bisphosphonates: clinical flowchart for long-term treatment and monitoring. GC, glucocorticoids (oral ≥ 7.5 mg prednisolone/day or equivalent); BP, bisphosphonate

1. Plan to prescribe oral bisphosphonates (alendronate, ibandronate and risedronate) for at least 5 years and then re-assess fracture risk. Longer durations of treatment, for at least 10 years, are recommended in the following men and women (Strong recommendation) (see Fig. 4):
  o Age ≥ 70 years at the time that the bisphosphonate is started
  o Who has a previous history of a hip or vertebral fracture(s)
  o Treated with oral glucocorticoids ≥ 7.5 mg prednisolone/day or equivalent
  o Who experiences one or more fragility fractures during the first 5 years of treatment (if treatment is not changed).
2. Plan to prescribe an annual intravenous bisphosphonate (i.e., zoledronate) for at least 3 years and then re-assess fracture risk. Longer durations of treatment, for at least 6 years, are recommended in the following men and women (Strong recommendation) (see Fig. 5):
  o Age ≥ 70 years at the time that the bisphosphonate is started
  o Who has a previous history of a hip or vertebral fracture(s)
  o Treated with oral glucocorticoids ≥ 7.5 mg prednisolone/day or equivalent
  o Who experiences one or more fragility fractures during the first 3 years of treatment (if treatment is not changed).
3. If a new fracture occurs after bisphosphonate treatment is discontinued, reassess using FRAX and restart treatment (Strong recommendation).
4. If bisphosphonate treatment is discontinued and no new fracture occurs, reassess using FRAX after 18 months for risedronate and ibandronate, 2 years for alendronate, and 3 years for zoledronate to inform whether treatment should be restarted (Strong recommendation).

Bisphosphonate therapy is associated with rare but serious adverse events, notably atypical femoral fractures (AFFs) and osteonecrosis of the jaw (ONJ). Defining the optimal duration of bisphosphonate therapy attempts to ensure that the benefit in fracture risk reduction outweighs the small risk of AFFs and ONJ at all time points through patient management. Bisphosphonates are retained long term in bone, allowing the beneficial effects to persist for some time after cessation of treatment administration. This has raised the possibility that some patients may benefit from a period of treatment to restore the benefit/risk balance [258]; (Evidence level IIa), in which treatment is stopped after some years and the need for reinstitution of therapy is subsequently reassessed. Treatment review in patients taking bisphosphonates is, therefore, critical [259] and each patient must be assessed individually to assess relative risks and benefits; there is no standard policy for ‘all patients’ [215]; (Evidence level IIa). Because pivotal clinical trials have mostly been limited to a duration of 3 years, recommendations for longer term use and for pauses in treatment are based on limited evidence from extension studies in postmenopausal women [260, 261]; (Evidence level IIa). There is currently no evidence on which to base specific recommendations for men.

Withdrawal of bisphosphonate treatment is associated with decreases in BMD and increased bone turnover after 2–3 years for alendronate [262, 263]; (Evidence level Ib), and 1–2 years for ibandronate and risedronate [264, 265]; (Evidence level Ib). In the case of zoledronate, withdrawal after 3 years’ treatment is associated with only a small decrease in BMD after a further 3 years without treatment [266]; (Evidence level Ib). Comparison between offset of alendronate and zoledronate at 3 years showed alendronate-treated patients had greater reductions in total hip BMD and greater rises in PINP, despite a longer treatment exposure with alendronate, supporting a more rapid offset of drug effect than with zoledronate [267]; (Evidence level IIb).

In the Fracture Intervention Trial Long-term extension study of alendronate (FLEX), there were significantly fewer clinical vertebral fractures in women previously treated with alendronate for 5 years who continued with alendronate for 5 more years than in those assigned to placebo after 5 years of alendronate [263]; (Evidence level Ib). In the Health Outcomes and Reduced Incidence with Zoledronate once yearly (HORIZON) study extension, the risk of morphometric vertebral fractures was significantly lower in women continuing on zoledronate for 3 years after the initial 3 years of therapy when compared to those switched to placebo [266]; (Evidence level Ib). Post-hoc analyses from the alendronate and zoledronate extension studies suggest that women most likely to benefit from long-term bisphosphonate therapy are those with low hip BMD (*T*-score <  − 2.0 in FLEX and ≤  − 2.5 in HORIZON), those with a prevalent vertebral fracture, and those who sustained one or more incident fractures during the initial 3 or 5 years of treatment [82]; (Evidence level Ib). Older age was also associated with increased fracture risk after discontinuation of alendronate therapy [268]; (Evidence level Ib).

## Reassessment of fracture risk in individuals on osteoporosis drug treatment

### Recommendations

5. Review treatment adherence in men and women who sustain a fragility fracture whilst on drug treatment, (poor adherence is when less than 80% of treatment has been taken correctly) and investigate for secondary causes of osteoporosis (Strong recommendation).
6. Fracture risk assessment in patients receiving drug treatment should be performed using FRAX with BMD, with arithmetic adjustments to FRAX probabilities to take account of additional clinical risk factors. If the FRAX-derived fracture probability exceeds the intervention threshold, drug treatment should be continued (Strong recommendation).
7. If biochemical markers of bone turnover indicate relapse from suppressed bone turnover and/or BMD has decreased following bisphosphonate withdrawal, consider resumption of drug treatment (Conditional recommendation).
8. After 10 years of bisphosphonate treatment, patient management should be considered on an individual basis (Conditional recommendation).

Stopping osteoporosis treatment, be it with bisphosphonate or denosumab, is associated with an increased risk of fragility fracture, such that routine cessation of anti-resorptive therapy (so called ‘drug holidays’) is not supported by a review of the evidence [215]; (Evidence level IIa). Reassessment of fracture risk in treated individuals can be performed using FRAX with femoral neck BMD [149]; (Evidence level IIb). The NOGG intervention thresholds can then be used to guide the decision as to whether treatment can be stopped for a period of time (Figs. 4 and 5). Whereas FRAX cannot be used to assess treatment response [149]; (Evidence level IIb) it does have a role in reassessing current fracture risk to determine the need to continue or to discontinue treatment. Detection of the offset of drug effect, using BMD and bone turnover changes, potentially provides information to influence clinical management. However, there are presently no definitive data that link a potential threshold change in BMD or bone turnover markers during drug offset to clinically meaningful changes in fracture risk.

## Rare adverse effects of long-term bisphosphonate and denosumab treatment

### Recommendations

9. During bisphosphonate or denosumab therapy, encourage all patients to maintain good oral hygiene, receive routine dental check-ups, and report any oral symptoms such as dental mobility, pain, or swelling (Strong recommendation).
10. In those with severe dental disease who require bisphosphonate or denosumab treatment, timely dental review and dental treatment by an appropriately experienced dental surgeon should be pursued before drug administration, bearing in mind drug treatment should be initiated as soon as possible after a fragility fracture; a multi-disciplinary team (MDT) approach to discuss individual needs is encouraged (Conditional recommendation).
11. During bisphosphonate or denosumab treatment, although ideally patients should minimise invasive dental procedures where possible, if indicated they can be carried out safely and successfully in most patients. When dental procedures are required, there are no data available to show whether treatment discontinuation reduces the risk of ONJ. Clinical judgment of the treating physician should guide the management plan of each patient based on individual benefit/risk assessment, ensuring patients continue to access routine dental care (Conditional recommendation).
12. During bisphosphonate or denosumab therapy, advise patients to report any unexplained thigh, groin, or hip pain, and if such symptoms develop, the femur should be imaged (by full length femur X-ray, isotope scanning or MRI) (Strong recommendation).
13. If an AFF is identified, image the contralateral femur (Strong recommendation).
14. All patients who develop an AFF should be referred to an osteoporosis specialist to guide management of future bone health (Strong recommendation).
15. In patients who develop an AFF, discontinue bisphosphonate or denosumab treatment (Conditional recommendation).

### Osteonecrosis of the jaw (ONJ)

*Osteonecrosis* occurs only very rarely in patients receiving bisphosphonate or denosumab therapy for osteoporosis. The estimated incidence in those receiving bisphosphonates is 10–100/100,000 person-years of exposure in clinical trials. Risk factors for ONJ include poor oral hygiene, dental disease, dental interventions, smoking, cancer, chemotherapy and/or glucocorticoid therapy [269, 270]; (Evidence level IIa). The incidence of ONJ is substantially greater with the higher doses of bisphosphonates or denosumab that are used to treat patients with skeletal metastases. The Scottish Dental Clinical Effectiveness Programme has produced guidance on oral health management in patients taking anti-resorptive medication [271]. *Osteonecrosis of the external auditory canal* after bisphosphonate treatment has been described very rarely in case reports, with patients presenting with ear symptoms including chronic ear infections. Possible risk factors include steroid use and chemotherapy and/or local risk factors such as infection or trauma [272]; (Evidence level IV).

### Atypical femoral fractures (AFF)

Atypical femoral fractures, mainly of the subtrochanteric and diaphyseal regions of the femoral shaft, have been reported rarely in patients taking bisphosphonates or denosumab for osteoporosis. Asian race, femoral bowing, and glucocorticoid use have been identified as risk factors [273]. In a recent review by the ASBMR Task Force on the management of osteoporosis in patients on long-term bisphosphonates, a systematic search of the literature revealed that the absolute risk was consistently low, ranging between 3.2 and 50 cases/100,000 person-years of exposure [274, 275]; (Evidence level IV). This estimate appeared to double with prolonged duration of BP use (> 3 years, median duration 7 years), and declined with discontinuation [274, 275]; (Evidence level IV), [276]; (Evidence level IIa). In a nationwide cohort study from Denmark, use of alendronate in excess of 10 years was associated with a 30% lower risk of hip fracture and no increase in the risk of fractures of the subtrochanteric femur and femoral shaft, supporting an acceptable risk–benefit balance in terms of fracture outcomes [277]; (Evidence level IIb). *Atypical femoral fractures* are often bilateral, associated with prodromal pain, and tend to heal poorly. Prodromal pain can be felt in the thigh, groin, or hip for days, weeks, or months before fracture. Discontinuation of bisphosphonate or denosumab therapy is advised in patients who develop an atypical fracture; weight-bearing activity should be restricted, adequate calcium and vitamin D should be ensured, and alternative treatment options considered where appropriate. Surgical treatment with intramedullary nailing is often recommended [274, 275]; (Evidence level IV).

There is a lack of good quality evidence on the medical management of bone health following an AFF. However, a recent international expert consensus document supported by a systematic review proposed practical measures to help in patient management [278]; (Evidence level IV). Following an AFF, if the risk of fragility fracture is low, further pharmacological bone treatments can be avoided. If fracture risk is high and bilateral surgical fixation of fractures has been performed, consider the use of teriparatide. If unilateral or no surgical intervention has taken place, consider teriparatide, romosozumab, raloxifene, or HRT. The potential utility of teriparatide as an adjunct to healing following AFF has been examined. There is no evidence that teriparatide enhances healing of AFFs, but limited data show a tendency towards faster healing in surgically managed AFFs (complete and incomplete). However, in AFFs managed conservatively, there was no suggestion of improved fracture healing with teriparatide [278]; (Evidence level IV). The benefits versus risks of using bisphosphonates or denosumab after AFF should be carefully examined if these options are considered, taking into consideration prior unilateral or bilateral nailing, the use of an anabolic agent post AFF, together with the overall clinical situation and fracture risk (Evidence level IV).

## Glucocorticoid-induced osteoporosis

### Recommendations

15. Because bone loss and increased fracture risk occur early after initiation of oral glucocorticoids, bone-protective treatment should be started in the following people, at the same time as glucocorticoid therapy without waiting for bone density assessment, which should follow later (Strong recommendations):
  - anyone with a prior fragility fracture,
  - women age ≥ 70 years,
  - postmenopausal women, and men age ≥ 50 years, prescribed high doses of glucocorticoids, i.e., ≥ 7.5 mg/day of prednisolone or equivalent over 3 months (*N.B*., this is equivalent to ≥ 30 mg/day of prednisone for 4 weeks over 3 months).
  - postmenopausal women, and men age ≥ 50 years, with a FRAX probability of major osteoporotic fracture or of hip fracture exceeding the intervention threshold.
16. Oral bisphosphonates (alendronate or risedronate) or intravenous zoledronate are the most cost-effective first-line drug options for bone protection. Denosumab is an alternative option. Teriparatide can be a first-line drug option in those at very high fracture risk (Strong Recommendation).
17. Adequate calcium intake should be achieved through dietary intake if possible, with the use of supplements if necessary. An adequate vitamin D status should be maintained, using supplements if required (Strong Recommendation).
18. If glucocorticoid therapy is stopped, withdrawal of bone-protective therapy may be considered at the same time, provided on re-assessment of fracture risk using FRAX, the probabilities of both major osteoporotic fracture and of hip fracture lie below the intervention threshold (Strong Recommendation).
19. If glucocorticoids are continued long term, bone protection should be maintained in the majority of cases (Strong Recommendation).
20. Patients starting medium or low dose oral glucocorticoid therapy who have a FRAX probability near to, but below the intervention threshold, should have FRAX with BMD reassessed 12–18 months after starting glucocorticoid therapy (Conditional recommendation).

Abaloparatide and romosozumab are further options for treatment if their therapeutic indication is fulfilled, i.e., in postmenopausal women at very high fracture risk. Bone protective therapy may be appropriate in some premenopausal women and younger men, particularly in individuals with a previous history of fracture, or those receiving high doses of glucocorticoids (≥ 7.5 mg/day of prednisolone or equivalent over 3 months). Caution is advised when prescribing drug treatment in women of childbearing age. Referral of complex cases to secondary care is often necessary. Although guidance on the prevention and management of glucocorticoid-induced osteoporosis has been developed in many countries, there is evidence that in the UK osteoporosis risk assessment and management are still inadequate in long-term users of oral glucocorticoids [279]; (Evidence level IIIb). Bone loss and increased fracture risk occur rapidly after initiation of oral glucocorticoid therapy and increase with the dose of glucocorticoids [67, 280]. The increase in fracture risk is seen for vertebral and non-vertebral fractures, including hip fractures, and is partially independent of BMD [68]; (Evidence level Ia).

Approval for the use of bone protective therapy to prevent osteoporosis in people receiving oral glucocorticoids was based mainly on BMD bridging studies carried out as part of Phase III randomised controlled trials with bisphosphonates [191, 196, 203, 281, 282]. Subsequently, approval has been given for denosumab using the same methodology [206]. Fracture prevention has not been considered as an efficacy end-point in most trials. However, although not a primary end-point, in an 18-month randomised controlled trial extended to 36 months comparing teriparatide with alendronate, significantly fewer subjects in the teriparatide group had vertebral fractures compared with the alendronate arm [244], but with no benefit on non-vertebral fractures. This protection against vertebral fractures was confirmed in a recent meta-analysis, which showed that co-prescription of teriparatide, alendronate, risedronate, or denosumab with glucocorticoids could reduce the incidence of vertebral fractures, with further evidence of a reduction in non-vertebral fracture rates with alendronate or teriparatide (Table 6) [283]; (Evidence levels Ia & Ib).

**Table 6:** Effect of approved interventions for glucocorticoid-induced osteoporosis on BMD and fracture risk

Bone protective therapy	Spine BMD	Hip BMD	Vertebral fracture	Non-vertebral fracture	Evidence of superiority for spine and/or hip BMD
Alendronate	Ib	Ia	Ia	Ia	Inferior to teriparatide (Ib)
Risedronate	Ib	Ia	Ia	NAE	Inferior to zoledronate (Ia)
Zoledronate	Ib	Ib	Ia	NAE	Superior to risedronate (Ib)
Denosumab	Ib	Ia	Ia	NAE	Superior to bisphosphonates (IIa)
Teriparatide	Ib	Ib	Ia	Ia	Superior to alendronate (Ib)

*NAE* no available evidence

Considering the increased fracture risk associated with higher glucocorticoid doses, FRAX assessment provides fracture probabilities based on both an average dose of oral prednisolone (2.5–7.5 mg/day or its equivalent) and a higher dose (≥ 7.5 mg/day or its equivalent). Individuals taking an average dose of prednisolone < 2.5 mg/day will have lower fracture risk, and the average adjustments over all ages in postmenopausal women and men aged ≥ 50 years are shown in Table 7 [28]; (Evidence level IIb). For very high doses of glucocorticoids, i.e., > 20 mg/day prednisolone or its equivalent, greater upward adjustment of fracture probability is required [67]; (Evidence level IIa).

**Table 7:** Adjustment of FRAX derived fracture probability estimates according to daily dose of prednisolone

Dose	Prednisolone equivalent dose (mg/day)	Average adjustment to hip fracture probability	Average adjustment to major osteoporotic fracture (MOF) probability
Low	< 2.5	− 35%	− 20%
Medium	2.5–7.5	None	None
High	≥ 7.5	+ 20%	+ 15%

When the UK FRAX model is used and the glucocorticoid box is filled, 2 points appear on the NOGG graphs, one for medium dose and one for high dose (all defined as above). The assessment thresholds (fracture probabilities for BMD testing) and intervention thresholds (fracture probabilities for therapeutic intervention) are then used in the same way as described for postmenopausal women and older men.

## Men receiving androgen-deprivation therapy

### Recommendations

The NOGG supports the guideline published by Brown et al. 2020 [284].

21. All men starting androgen deprivation therapy (ADT) should have their fracture risk assessed using FRAX, considering ADT use as a secondary cause of osteoporosis, with BMD measured where available (Strong recommendation).
22. Consider referring men, with high fracture risk requiring drug treatment, to secondary care for assessment and initiation of treatment with bisphosphonates or denosumab (Conditional recommendation).
23. Men with FRAX probability near to, but below the intervention threshold, and patients going on to additional systemic therapies (particularly those requiring glucocorticoids), should have FRAX with BMD reassessed 12–18 months after starting ADT (Conditional recommendation).

There is no evidence that skeletal metabolism in men differs fundamentally from that of women [285]. However, secondary causes of osteoporosis are common in men and amongst these hypogonadism is prominent [286]. Androgen deprivation therapy (ADT), used primarily in the treatment of older men with prostate cancer, is frequently associated with hypogonadism. Osteoporosis caused by ADT is associated with rapid loss of BMD within 6–12 months of initiation of ADT [287]; (Evidence level Ic). There is a significant increase in fracture risk in men with prostate cancer in the 5 years following the initiation of ADT when compared to those not receiving ADT [288]; (Evidence level Ic). Bisphosphonates and denosumab are effective drug treatments for preventing BMD loss in men with prostate cancer taking ADT, although effects on fracture risk have not been demonstrated. Exercise programmes are a less effective alternative which are insufficient in isolation [289]; (Evidence level Ib).

In a systematic review and network meta-analysis, all evaluated treatments for ADT-induced bone loss, which included bisphosphonates and selective oestrogen receptor modulators (SERMs), were effective in improving BMD compared to placebo. However, zoledronate generated greater improvements in BMD compared to other drug treatments at all bone density sites, except for risedronate, which had better BMD improvement compared to zoledronate at the femoral neck site in one small study [290]; (Evidence level IIa). A recent UK consensus statement on prostate cancer treatment-induced bone loss concluded that fracture risk should be calculated using FRAX, considering ADT use as a secondary cause of osteoporosis and including BMD where available and practical. BMD should always be assessed where calculated fracture risk is close to the NOGG intervention threshold. Those with FRAX probability near to but below the intervention threshold and patients going on to additional systemic therapies should have FRAX with BMD repeated after 12 to 18 months [284]; (Evidence level IIa).

## Women receiving aromatase inhibitor therapy

### Recommendations

24. All women starting aromatase inhibitor (AI) therapy should have their fracture risk assessed using FRAX, considering AI use as a secondary cause of osteoporosis, including BMD measurement where practical (Strong recommendation).
25. Women with high fracture risk should be commenced on drug treatment to prevent osteoporosis and fracture, with bisphosphonates or denosumab (Strong recommendation).
26. Women with a FRAX probability near to, but below the intervention threshold, and patients going on to additional systemic therapies (particularly those requiring glucocorticoids), should have FRAX with BMD reassessed 12–24 months after starting AI therapy (Conditional recommendation).
27. If adjuvant high-dose bisphosphonate therapy is used as part of breast cancer management, consider assessing fracture risk at the end of this bisphosphonate therapy, particularly if AI therapy continues (Conditional Recommendation).

The use of aromatase inhibitors (AI) in postmenopausal women induces bone loss at an average rate of 1–3% per year at sites rich in trabecular bone. Bone loss is more marked in young women with treatment-induced ovarian suppression, losing an average of 7–8% per annum [291]; (Evidence level IIa). In case–control studies, the incidence of fracture in women with breast cancer treated with AIs is reported to be around 18–20% after 5 years follow-up [292]. NICE guidance on the management of early breast cancer, which recognises the excess risk of osteoporosis with the use of AIs, recommends a baseline DXA scan to assess BMD at the time of initiation of AI therapy [293]; (Evidence level IV). International Consensus Position Statements suggest that fracture risk should be assessed, although the consideration of AI use as a secondary cause of osteoporosis in FRAX may not adequately estimate fracture risk [292, 294]; (Evidence level IIa) with drug treatment to prevent bone loss and fractures recommended in those with a *T*-score of less than − 2, or less than − 1.5 with 1 additional risk factor, or in those with 2 or more risk factors (without BMD). Drug treatment should be a bisphosphonate (oral or parenteral) or denosumab, used in the doses as for postmenopausal osteoporosis. Denosumab and zoledronate both lead to significant gains in BMD at the spine and hip in postmenopausal women with breast cancer receiving AIs, and both denosumab and risedronate have been shown to reduce fracture risk [295]; (Evidence level Ia).

## Management of symptomatic osteoporotic vertebral fractures

### Recommendations

1. Administer analgesia orally rather than parenterally whenever possible. *P*ain should be regularly reviewed, and analgesia titrated up or down according to pain intensity and side effects, with use of the weakest effective agent for the shortest possible time (Strong recommendation).
2. Avoid use of non-steroidal anti-inflammatory drugs (NSAIDs) in older people, but, if used, co-prescribe a proton-pump inhibitor, and monitor for gastrointestinal, renal and cardiovascular side-effects (Strong recommendation).
3. Prescribe appropriate laxative therapy, such as the combination of a stool softener and a stimulant laxative, whenever opioid therapy is used in older people (Strong recommendation).
4. It is recommended that exercise programmes following vertebral fracture include progressive muscle strengthening activity, including back extensor muscle strengthening and/or endurance exercise (Strong recommendation).
5. When a patient is in pain, it may be advisable to initially perform exercise for back extensors in an unloaded position (Conditional recommendation).
6. Provide clear and prompt guidance on how to adapt movements involved in day-to-day living, including how exercises can help with posture and pain, to patients with painful vertebral fractures (Strong recommendation).
7. Ensure prompt secondary fracture prevention is started following a fracture, with follow-up through fracture liaison services for all postmenopausal women, and men aged 50 years and older, with a newly diagnosed vertebral fracture (Strong recommendation).

Vertebral fractures can cause acute and chronic pain, height loss, spinal deformity and altered body shape, functional impairment, and reduced health-related quality of life [14]; (Evidence level Ia). Analgesia for acute pain is important to allow restoration of function and mobility but must be used safely [296–298]; (Evidence level IIa). In patients admitted to hospital, salmon calcitonin given for up to 4 weeks (50–100 IU daily given subcutaneously or intramuscularly) has been shown to be an effective adjunctive analgesic for pain experienced at rest or when walking, associated with acute (within 10 days of) vertebral fracture [299]; (Evidence level IIa). However, side effects (mainly flushing and gastro-intestinal disturbance) are common. Long-term use may be associated with an increased risk of cancer [300]. There is no evidence that salmon calcitonin is an effective treatment for chronic pain associated with vertebral fractures [299]; (Evidence level Ia). Of note, in the SPC, calcitonin is indicated for the prevention of acute bone loss due to sudden immobilisation such as in patients with recent osteoporotic fractures, rather than for the management of pain. A single, small, randomised double-blind, controlled trial found 30 mg intravenous pamidronate, given within 21 days of acute vertebral fracture, to be more effective than placebo in reducing pain [301]; (Evidence level IIb). Of note in the SPC, pamidronate is indicated for the treatment of conditions associated with increased osteoclast activity, rather than for the management of pain. Physiotherapist supervised exercise following vertebral fracture improves pain and physical performance [302]; (Evidence level Ib). In the presence of pain, it may be advisable to initially perform exercise for back extensors in an unloaded position, such as supine [303]; (Evidence level Ia).

Combining exercise with physiotherapy-delivered education and guidance can reduce fear of falling and improve psychological symptoms associated with vertebral fractures [171, 304]; (Evidence level Ia). For patients with painful vertebral fractures, there is low quality evidence suggesting that spinal bracing using soft or rigid external orthoses for 2 h a day over 6 months may improve pain and trunk muscle strength [303]. There is currently no evidence that bracing with soft or rigid external orthoses improves fracture healing [305]. Hence, routine use of bracing for the treatment of acute or subacute vertebral fractures cannot be recommended (Evidence level Ia). The current evidence does not support the routine use of percutaneous vertebroplasty or balloon kyphoplasty for the treatment of painful osteoporotic vertebral fractures, as these procedures do not show consistent patient benefit [303, 306]; (Evidence level Ia). In older women with vertebral fractures and chronic back pain stable for 6 months or more, a small randomised controlled trial has shown electrical nerve stimulation, administered as inferential therapy or horizontal therapy 5 days a week for 2 weeks, can improve pain over 14 weeks [307]; (Evidence level IIb). Patients with a recent vertebral fracture have a high imminent risk of further fragility fracture [64]; (Evidence level IIb). If a vertebral fracture is associated with impending or existing neurological deficits, urgent referral to spinal surgical services is indicated.

## Models of care for fracture prevention

### Recommendations

1. Multidisciplinary, coordinator-based FLS are recommended to systematically identify men and women with fragility fractures, facilitating timely assessment of fracture and falls risk, and where appropriate, tests to exclude secondary causes of osteoporosis, radiological investigation including BMD testing, and initiation of pharmacological and non-pharmacological interventions to reduce risk of falls and fractures (Strong recommendation).
2. FLSs should include embedded local audit systems supported by a clinical fracture database to enable monitoring of care provided to fracture patients [e.g., Royal College of Physicians FLS-Database]; (Strong recommendation).
3. FLSs should employ a range of case-finding strategies to identify all inpatients and outpatients with fragility fractures (Strong recommendation).
4. Diagnostic imaging services should routinely evaluate the spine in all imaging of postmenopausal women, and men aged ≥ 50 years, in which the spine is visualised, and report vertebral fractures using standardised methods (Strong recommendation).
5. Patients recommended drug treatment for osteoporosis should be offered tailored information about osteoporosis and its treatments, and further medication reviews to support adherence and to discuss alternative treatments if unacceptable adverse events arise or adherence is difficult (Strong recommendation).
6. Primary care clinicians should always have in mind the possibility of vertebral fracture in postmenopausal women and men aged ≥ 50 years who present with acute onset back pain, especially thoracic pain, if they have risk factors for osteoporosis (Strong recommendation).

### FLS models of care

Collaboration between primary care clinicians, secondary care physicians, orthopaedic surgeons, radiologists, and pharmacists, and between the medical and non-medical disciplines concerned, should underpin secondary fracture prevention programmes. Fracture Liaison Service (FLS) programmes reduce re-fracture rates and improve survival [308, 309] (Evidence levels Ia and IIb). The Department of Health and NHS RightCare both state that FLS should be provided for all patients sustaining a fragility fracture [310, 311], which aligns with the International Osteoporosis Foundation’s global Capture the Fracture® programme [312] and the Royal Osteoporosis Society (ROS) FLS Clinical Standards [313].

FLS should provide fully coordinated, intensive models of care for secondary fracture prevention. FLS models which provide identification, assessment and treatment initiation, or a treatment recommendation to primary care, are more clinically effective and cost-effective in improving patient outcomes than approaches that provide identification and/or patient alerts, and/or patient education only [314]; (Evidence Level Ia). The required approach is a FLS in which identification, assessment and osteoporosis treatment are all conducted within an integrated electronic health care network, overseen by a coordinator and utilising a dedicated database measuring performance [312, 314–316]; (Evidence level Ia). FLS that initiate pharmacological treatment, rather than making a treatment recommendation for primary care initiation, have higher rates of treatment initiation [315]; (Evidence level Ia). FLS should also initiate appropriate non-pharmacological interventions and communicate ongoing care effectively with primary care practitioners [313]. FLS should provide a coordinated programme with an integrated approach for falls and fracture prevention; all individuals with a fracture should be fully assessed for falls risk and appropriate interventions to reduce falls should be undertaken [317]. As risk of re-fracture is highest immediately after a fragility fracture, secondary fracture prevention assessment and intervention should be initiated as soon as possible and no later than 16 weeks post-fracture, as recommended by the Royal Osteoporosis Society [64, 313].

### FLS patient identification

FLSs need to employ a range of case finding strategies to identify both inpatients and outpatients with fragility fractures and people with vertebral fractures who are often undiagnosed. Reasons for non-identification of vertebral fractures include the absence of a fall as a trigger for investigation, absence of symptoms or attribution of symptoms to other causes. Furthermore, in patients who do have spinal imaging, use of ambiguous non-standardised terminology in imaging reports and failure to routinely evaluate the vertebrae captured in imaging of other body systems can both contribute to non-identification of vertebral fractures. The Royal Osteoporosis Society recommends that radiology services should establish local processes to ensure that the spine is routinely evaluated for the presence of vertebral fracture in all available imaging and that reports identifying vertebral fractures should be standardised, using the words ‘vertebral fracture’, are actionable and indicate future management [318]; (Evidence level IV).

Primary care plays an important role in case finding for osteoporotic fractures, particularly vertebral fractures, as acute onset back pain, especially thoracic pain, is a common presenting complaint. Targeted use of spinal imaging can help increase case identification, appropriate symptom management, and prompt secondary fracture prevention.

### Providing patient information and adherence support

Patients identified by any clinical service, to be in need of further intervention, should be offered an explanation of osteoporosis, the causes, consequences and how it can be managed with pharmacological and non-pharmacological interventions. When discussing pharmacological treatment, explanations should be offered for why drug treatment is recommended, the aims and benefits, common and/or severe side effects, the practicalities of taking the medicine and for how long it should be taken [319]; (Evidence level IV). The use of decision aids in osteoporosis to support communication of medicine risk–benefit has been shown to improve shared decision making, reduce decisional conflict and improve the accuracy of patient perceived fracture risk [320]; (Evidence level Ib). Information should be tailored to the needs of the patient to make it accessible and understandable, including the provision of written information [321].

To promote treatment adherence, healthcare professionals should elicit and address any beliefs and concerns associated with reduced adherence and establish realistic treatment expectations with the patient [319, 321]. No one type of intervention has been demonstrated to enhance medicines adherence in osteoporosis care, but multi-component models with active patient engagement have the most positive effects [322, 323]; (Evidence level Ia). FLS models with a greater number of patient interactions have demonstrated greater clinical effectiveness [316]; (Evidence level Ia). The NOGG supports the Royal Osteoporosis Society recommendation to follow up within 16 weeks and 52 weeks post-fracture, to review use of medications that increase the risk of falls and/or fracture, to ensure co-prescription of calcium and vitamin D with bone protective interventions where indicated, to review adverse effects and monitor adherence to therapy [313].

## Recommendations for training

### Recommendations

It is recommended that:

1. Training in personalised care, including shared decision making, is provided within all higher professional training curricula in relevant medicine and surgical specialities (Strong recommendation).
2. Training in osteoporosis and metabolic bone diseases is a clearly articulated component of each of the relevant medical and surgical specialities’ higher professional training curricula set out by the applicable medical and surgical Royal Colleges (Strong recommendation).
3. Primary care physicians have sufficient training in this area with efficient access to up-to-date evidence-based resources and guidelines, and continual professional development (CPD) opportunities to maintain and refine knowledge (Strong recommendation).
4. The management of osteoporosis is a component of training in all relevant allied health disciplines (Strong recommendation).
5. Training should be provided to Fracture Liaison Service personnel to achieve high-quality DXA performance and reporting (Strong recommendation).
6. Quality improvement training should be provided to healthcare personnel responsible for the delivery of Fracture Liaison and/or Osteoporosis Services (Strong recommendation).

The management of osteoporosis and fragility fracture risk is not subserved by any one specialty. The relevant medical and surgical specialties include general practice, rheumatology, orthopaedic surgery, endocrinology, metabolic medicine, renal medicine, geriatric medicine, and obstetrics and gynaecology. Furthermore, the care of patients with osteoporosis is the responsibility of multiple healthcare professionals, including nurses, physiotherapists, occupational therapists, pharmacists, and DXA operators. The multi-disciplinary nature of osteoporosis care offers opportunities for cross-speciality training. It is recognised that primary care is pivotal to the identification of the population at risk of fragility fractures as well as to the long-term management of patients with osteoporosis. It is important that primary care physicians have sufficient training in this area, with access to resources such as updated guidelines and online learning modules to refresh their knowledge.

Common to all healthcare roles is a need to provide personalised patient-centred care, a key commitment outlined by the NHS to be achieved by 2023/24. Personalised care is a partnership approach that helps people make informed decisions and choices about their health and wellbeing, working alongside clinical information [Personalised Care Institute 2020]. There is significant variability in the access to and quality of DXA services for established FLS worldwide. Despite two decades of training initiatives in osteoporosis densitometry, many centres are falling short of the standards of the IOF-ISCD Osteoporosis Essentials criteria [324].

Improving the quality of osteoporosis and fracture liaison services is about making health care delivery safe, effective, patient-centred, timely, efficient, and equitable. Quality improvement involves the use of a systematic and coordinated approach to solving a problem using specific methods and tools with the aim of bringing about a measurable improvement within a health care setting [325], and can be aided by the use of appropriate Toolkits (e.g. the Royal Osteoporosis Society Fracture Liaison Service Implementation Toolkit).

### Examples of appropriate training

i. Training in Personalised Care. Training in enhanced consultation skills including risk communication, shared decision making, applying principles of health literacy and how to communicate about osteoporosis is available at http://www.ifraptraining.co.uk. The Personalised Care Institute is a virtual organisation, accountable for setting the standards for evidence-based training in personalised care in England. The Personalised Care Institute Curriculum sets out the standards for training programmes to become accredited with the Personalised Care Institute. The Personalised Care Institute provides eLearning modules, for example, on Shared Decision Making. The curriculum is designed for health care personnel within primary and secondary care and community teams https://www.personalisedcareinstitute.org.uk.
ii. Training in Osteoporosis Management. The Royal Osteoporosis Society Fracture Prevention Practitioner Training is accredited for CPD by RCGP, RCP and RCN. The online training includes five foundation modules and then three advanced modules https://theros.org.uk/healthcare-professionals/courses-and-cpd/fracture-prevention-practitioner-training/. The Royal College of General Practice also provides a short e-Learning module on the diagnosis and management of osteoporosis https://elearning.rcgp.org.uk/course/info.php?id=233. Evidenced-based resources to support osteoporosis care are available at www.erohub.co.uk.
iii. Training in Musculoskeletal Pain Management. The Health Education England e-Learning for Healthcare Pain Management programme includes training on musculoskeletal pain which encompasses the assessment and management of osteoporotic vertebral fractures https://www.e-lfh.org.uk/programmes/pain-management/.
iv. Training in DXA conduct. The Royal Osteoporosis Society runs a National Training Scheme for Bone Densitometry. This online course provides a foundation in osteoporosis and DXA. All ROS course information is available here https://theros.org.uk/healthcare-professionals/courses-and-cpd/.

## Recommendations for commissioners of healthcare

In 2017, the National Falls Prevention Coordination Group with Public Health England (PHE). Produced a falls and fracture consensus statement and resource pack with the aim of reducing falls and fracture risk and improving management of fractures, including secondary prevention.

(https://www.gov.uk/government/publications/falls-and-fractures-consensus-statement). The guidance is aimed at local commissioning and strategic leads in England with a remit for falls, bone health and healthy ageing. Following this, NHS RightCare, working with PHE and the Royal Osteoporosis Society (ROS), developed a Falls and Fragility Fractures Pathway https://www.england.nhs.uk/rightcare/products/pathways/falls-and-fragility-fractures-pathway/) which defines three priorities that commissioners responsible for falls and fragility fractures should optimise as a priority: (i) falls prevention, (ii) detecting and managing osteoporosis and (iii) optimal support after a fragility fracture. The ROS has developed an online Fracture Liaison Service Implementation Toolkit (https://theros.org.uk/healthcare-professionals/fracture-liaison-services/implementation-toolkit/) designed to enable FLS Commissioning. In England, the move to Integrated Care Systems (ICS) provides an opportunity to embed enhanced pathways of care for patients at risk of fragility fracture, including imminent fracture risk [326], as part of routine service delivery, for example enabling direct referrals between different secondary care services to streamline patient care pathways.

Where healthcare funding is not delivered through a commissioning structure, the recommendations below apply to bodies providing healthcare funding and to local health boards. Thus, in Wales, these recommendations apply to the Welsh Government and to local health boards (that are funded directly from the Welsh Government) when setting their Integrated Medium-Term Plans (IMTPs). In Northern Ireland, health and social care are integrated and are the responsibility of the Department of Health. Health services are commissioned by the Health and Social Care Board (HSCB) through local commissioning groups from the five Health and Social Care Trusts. Thus, in Northern Ireland, these recommendations apply to the HSCB and to the five local commissioning groups.

### Recommendations

Based upon the evidence presented in this guideline, the NOGG makes the following recommendations to service leaders and/or commissioners of healthcare who:

1. Should recognise that fractures due to osteoporosis are a significant and growing public health issue with consequent high health and social care costs and ensure that fragility fractures are addressed explicitly in their local healthcare programmes (Strong recommendation).
2. Should ensure that local healthcare programmes address approaches to reduce the prevalence of avoidable risk factors for osteoporosis and fractures related to falls and poor bone health and, in so doing, make explicit the roles of both the NHS and other agencies (Strong recommendation).
3. Should ensure electronic patient health record systems have FRAX, and the link to the NOGG website, integrated to aid identification and treatment of those at risk of fragility fracture, and that electronic patient health record systems enable clear, and where possible automated, electronic communication between FLS and primary care teams (Strong recommendation).
4. Should put arrangements in place so that those at risk of osteoporotic fractures have the opportunity to receive appropriate investigation (e.g., fracture risk assessment, falls risk assessment, bone density measurement), lifestyle advice (e.g., about diet, exercise, and smoking) and bone protective drug therapy [NICE Quality Standards 149, 2017]. The latter includes the availability of parenteral drug therapies in primary care and community healthcare settings (Strong recommendation).
5. Should ensure that accurate, up-to-date consistent information about pharmacological drug interventions is widely available to postmenopausal women and men aged ≥ 50 years, their healthcare advocates and professional advisers, so that patients can make informed decisions about treatment and treatment adherence (Strong recommendation).
6. Integrated Care Systems (ICS) should specifically address the burden of fragility fractures on the local economy and ensure that Fracture Liaison Services are available for all patients who sustain a fragility fracture (Strong recommendation).
7. ICS should bring together local specialists, generalists, and other stakeholders, including patient representatives, to agree local treatment practices and referral pathways for the management of osteoporosis and prevention of fragility fractures. It is often helpful to identify a lead clinician in both primary and secondary care. The recommendations of this group should take account of local resources and relevant cost-effectiveness data. Local guidelines should be consistent with the evidence presented in this document. Once local guidelines have been agreed, they should be widely disseminated to relevant professionals and potential patients, and the necessary service changes made to allow the guidelines to be implemented. Implementation should be audited, and appropriate changes in practice should be instituted where standards are not met, with appropriate monitoring of compliance to guidelines thereafter (Strong recommendation).

## Review criteria for audit and quality improvement

### Quality standards for osteoporosis

1. Four quality standards for osteoporosis were produced by the National Institute for Health and Care Excellence (NICE) in 2017 (QS149) (https://www.nice.org.uk/guidance/qs149).
2. Seven quality standards for osteoporosis and the prevention of fragility fractures were produced by the Royal Osteoporosis Society in 2017 (https://theros.org.uk/media/0dillsrh/ros-op-standards-november-2017.pdf)
3. The Royal Osteoporosis Society has produced five clinical quality toolkits: DXA quality, Hip fracture, Vertebral fracture, Fracture Liaison Service (FLS) implementation, and virtual and telephone appointments (https://theros.org.uk/healthcare-professionals/clinical-quality-hub/clinical-quality-toolkits/).

### Primary care

4. Documentation of the proportion of postmenopausal women and men age ≥ 50 years registered with a general practice:
  - With a fracture code, who have been assessed to determine whether their fracture was a fragility (low-trauma) fracture.
  - With one or more risk factors for fragility fracture, who receive formal fracture risk assessment.
  - With a prior fragility fracture, who have had a DXA scan with the result recorded.
  - Calculated to be high or very high risk by FRAX assessment, who have been offered drug treatment.
  - With an incident hip fracture, those who receive pharmacological drug therapy for osteoporosis within 16 weeks of their fracture.
  - Who are prescribed pharmacological drug therapy for osteoporosis and who have had confirmed adherence to osteoporosis therapy within the last 12 months.
  - Who are prescribed pharmacological drug therapy for osteoporosis and have had a 5-year and 10-year review.
  - Who are prescribed denosumab, who have received timely (within 4 weeks of due date) follow-up injection.
  - Who are on oral glucocorticoids for ≥ 3 months and have had a fracture risk assessment.
  - With documented discussion of fracture risk assessment and a treatment decision.

### Fracture liaison services

5. The Royal Osteoporosis Society (ROS) published in 2019 six key standards for FLS with a corresponding timeline for the achievement of these six steps, with examples of audit and evidence [313]. This was followed by issuing the FLS Implementation Toolkit (https://theros.org.uk/healthcare-professionals/clinical-quality-hub/fracture-liaison-services/implementation-toolkit/).
6. The Royal College of Physicians FLS Database National Audit (https://www.rcplondon.ac.uk/projects/fracture-liaison-service-database-fls-db) is commissioned by the Healthcare Quality Improvement Partnership (HQIP) as part of the Falls and Fragility Fracture Audit Programme. The FLS-DB is included in the HQIP listing for national audits that must be reported in each English hospital trust’s Quality Account and is required by the Welsh Government for all Health Boards in Wales. These form part of the National Clinical Audit Patient Outcomes Programme. All FLS sites that treat fractures are eligible to participate. The FLS-DB sets out 11 Key Performance Indicators (KPIs) which are designed to measure performance against technology assessments, guidance on osteoporosis and clinical standards for FLSs from the NICE, the ROS and NOGG.
7. The International Osteoporosis Foundation (IOF) Capture the Fracture Best Practice Framework outlines 13 standards for FLS delivery with criteria and targets specified for bronze, silver, or gold levels of achievement (https://www.capturethefracture.org/best-practice-framework).

### DXA reporting

8. The ROS published in 2019 six quality standards for DXA reporting with a corresponding audit template [51].

## Summary of main recommendations

This guideline summary addresses the assessment, diagnosis, and current treatments for osteoporosis, including recommendations to prevent fragility fractures. It applies to postmenopausal women and to men aged 50 years or older.

### Concerning assessment of fracture risk in postmenopausal women, and men age ≥ 50 years:

1. Conduct a FRAX assessment in people with a clinical risk factor for fragility fracture.
2. Measure BMD in people with intermediate fracture risk by FRAX (*amber*) to refine the estimate of 10-year risk.
3. Measure BMD in people with high and very high fracture risk by FRAX (*red*) to guide drug choice and provide a baseline for BMD monitoring.
4. Consider imaging to look for a vertebral fracture in people with acute onset back pain who have risk factors for osteoporosis, and/or in people with a history of ≥ 4 cm height loss, kyphosis, recent or current long-term oral glucocorticoid therapy, or a BMD *T*-score ≤  − 2.5.
5. Assess falls risk in patients with osteoporosis and/or fragility fractures and offer those at risk an exercise programme to improve balance and muscle strength.

### Regarding drug treatment to prevent fractures in postmenopausal women, and men age ≥ 50 years:

6. Offer drug treatment to people at high and very high risk of fracture.
7. If BMD measurement is not practical (e.g*.* due to frailty), use the online NOGG intervention thresholds based on FRAX, to guide treatment decisions.
8. Consider, particularly in older people, drug treatment in those with a prior and/or recent fragility fracture.

### When selecting drug treatments to prevent fractures in postmenopausal women, and men age ≥ 50 years:

9. Consider the level of fracture risk, any additional clinical risk factors, patient choice, and the cost-effectiveness of treatment when deciding on a particular drug treatment.
10. Start treatment promptly following a fragility fracture, because the risk of re-fracture is highest immediately after a fracture and the risk remains elevated.
11. Consider referral of very high-risk patients to an osteoporosis specialist in secondary care for assessment and consideration of parenteral treatment (some may need first-line anabolic drug treatment, especially if multiple vertebral fractures). Indications of very high risk include the presence of important risk factors, including a recent vertebral fracture (within the last 2 years), ≥ 2 vertebral fractures (whenever they have occurred), BMD *T*-score ≤  − 3.5, treatment with high-dose glucocorticoids (≥ 7.5 mg/day of prednisolone or equivalent over 3 months); the presence of multiple clinical risk factors, particularly with a recent fragility fracture indicating high imminent risk of re-fracture; or other indicators of very high fracture risk, including as defined by FRAX.
12. In other patients for whom treatment is indicated, offer antiresorptive therapy with oral bisphosphonates (alendronate or risedronate) or intravenous zoledronate, or in postmenopausal women age ≤ 60 years, hormone replacement therapy.
13. Consider alternative treatment options if first-line bisphosphonates are unsuitable or not tolerated; denosumab, ibandronate, raloxifene, strontium ranelate, teriparatide, abaloparatide, or romosozumab.
14. Following treatment with an anabolic agent (teriparatide, abaloparatide or romosozumab), start alendronate, zoledronate, or denosumab without delay.

### When postmenopausal women, and men age ≥ 50 years, have started drug treatment:

15. Regularly review patients‘ tolerance of, and adherence to, oral drug treatments.
16. Remember long-term treatment is often required because osteoporosis is a long-term condition for which there is currently no cure.
17. Plans to prescribe oral bisphosphonates *for at least* 5 years, or intravenous bisphosphonates for at least 3 years and then re-assess fracture risk. Longer durations of treatment will be needed in those who are older (age ≥ 70 years), have had a hip or vertebral fracture, are on high-dose oral glucocorticoids (≥ 7.5 mg/day of prednisolone or equivalent over 3 months), or have a further fragility fracture during osteoporosis treatment. In lower risk patients, a temporary treatment pause of 18 to 36 months can be considered after 5 years’ oral bisphosphonate or 3 years’ intravenous bisphosphonate (see clinical flowcharts on p.50 and p.51).
18. Before starting denosumab, ensure the long-term treatment plan considers the potential need to stop denosumab and how this would be managed.
19. Do not stop denosumab treatment without a plan for subsequent anti-resorptive therapy, where renal function permits.
20. Repeat fracture risk assessment after any new fracture, regardless of when this occurs.
21. Reassess fracture risk 18 months to 3 years after pausing drug treatment.

### When postmenopausal women, and men age ≥ 50 years, are treated with oral glucocorticoids:

22. If starting ≥ 7.5 mg/day prednisolone or equivalent for the next 3 months, start bone protective treatment at the same time (without waiting for a DXA scan, which can follow later).
23. Offer antiresorptive therapy with oral bisphosphonates (alendronate or risedronate) or intravenous zoledronate, and in those at very high risk of vertebral fracture, refer for consideration of anabolic therapy.
24. Consider denosumab as an alternative treatment option.

### When advising on lifestyle and dietary measures:

25. Recommend a healthy, balanced diet, moderation of alcohol consumption and avoidance of smoking.
26. Ensure a sufficient dietary calcium and vitamin D intake and supplement these as necessary.
27. Encourage a combination of regular weight-bearing and muscle strengthening exercise.

### Regarding fracture prevention services:

28. Patients who sustain a fragility fracture should have access to a multidisciplinary, coordinator-based Fracture Liaison Service (FLS) which enables timely fracture and falls risk assessment, investigation, treatment, and monitoring.
29. Ensure that diagnostic imaging services routinely evaluate the spine in all imaging of postmenopausal women and men aged ≥ 50 years in which the spine is visualised and report vertebral fractures using standardised methods.

### When a postmenopausal woman, or a man age ≥ 50 years has a symptomatic osteoporotic vertebral fracture:

30. Consider referral to an exercise programme which provides progressive muscle strengthening activity, including back extensor muscle strengthening and/or endurance exercise.
31. Investigate for underlying causes of fragility fracture.
32. Start treatment promptly to reduce the risk of further fractures.

The evidence presented in this guideline underpins a further series of recommendations made for leaders and commissioners of healthcare services, as well as criteria for audit and quality improvement in primary and secondary care settings.

## Appendix 1

**List of stakeholders from 2021**

Association for Clinical Biochemistry and Laboratory Medicine.

Bone Research Society.

British Geriatrics Society.

British Orthopaedic Association.

British Orthopaedic Research Society.

British Menopause Society.

British Society for Rheumatology.

European Calcified Tissues Society.

European Society for Clinical and Economic Aspects of Osteoporosis, Osteoarthritis and Musculoskeletal Diseases.

International Osteoporosis Foundation.

Osteoporosis 2000.

Osteoporosis Dorset.

Primary Care Rheumatology and Musculoskeletal Medicine Society.

Royal College of Physicians.

Royal Osteoporosis Society.

Royal Pharmaceutical Society.

Society for Endocrinology.

The Nutrition Society.

## Appendix 2 Grading of evidence

*Levels of evidence for studies of intervention*

la   From systematic review and meta-analysis of randomised controlled trials (RCTs).

Ib   Individual RCT(s) (with narrow confidence intervals).

IIa  Systematic review of at least one non-randomised controlled trial or well-designed cohort study.

IIb  Individual cohort study or low quality RCTs.

IIIa Systematic review of at least one case-controlled study.

IIIb Individual case-control study.

IV   Expert committee reports or opinions and/or clinical experience of authorities, case series (and poor-quality cohort and case-control studies).

*Levels of evidence for validity of candidate risk factors*

Ia Systematic reviews or meta-analysis of level I studies with a high degree of homogeneity.

Ib Systematic reviews or meta-analysis with moderate or poor homogeneity.

Ic Level I studies (with appropriate populations and internal controls.

IIa Systematic reviews or meta-analysis of level II studies.

IIb Level II studies (in appropriate population or lacking an internal control).

IIIa Systematic reviews or meta-analysis of level III studies.

IIIb Case-control studies.

IV  Evidence from expert committees without explicit critical scientific analysis or that based on physiology, basic research or first principles.

Of note, FRAX risk factors are all grade A or B according to evidence for reversibility of risk [75]

*Grading of recommendations*

Recommendations follow the Grading of Recommendations Assessment, Development, and Evaluation GRADE binary classification of recommendations as either strong or conditional (also known as discretionary or qualified recommendations) [327]. Recommendations have been made after assessment of [328]:

1. The balance between desirable and undesirable effects -—The larger the difference between the desirable and undesirable effects, the more likely a strong recommendation is warranted.
2. The quality of evidence - the higher the quality of evidence, the more likely a strong recommendation is warranted.
3. Values and preferences - the more variability/uncertainty in values and preferences, the more likely a conditional recommendation is warranted.
4. Costs (resource allocation) - the higher the costs of an intervention (i.e., the more resources consumed), the more likely a conditional recommendation is warranted.

For example, a strong recommendation applies where the clinician considers that most people ought to receive the intervention, or where adherence to the recommendation could be used as a performance or quality indicator and that deviation from this recommendation would prompt documentation of a clinician’s rationale. NICE suggests using ‘offer’ (or similar action wording such as ‘measure’, ‘advise’, ‘commission’ or ‘refer’) when describing a strong recommendation [329].

A conditional recommendation applies where the clinician examines the evidence and prepares to discuss this with the patient together with the patient’s values and preferences, or where documentation of the discussion of the pros and cons of an intervention is the indicator of quality, rather than the course of action itself. NICE suggests using wording such as ‘consider’ when describing conditional recommendations. Where insufficient evidence is available or the evidence available is equivocal, recommendations are not made.

## Appendix 3

See Table 8.

**Table 8:** The quality of systematic reviews and meta-analyses used in the formulation of 2021 recommendations was assessed using AMSTAR2 (https://amstar.ca/Amstar-2.php).

Topic	Reference	Type of study	AMSTAR2 grading	Reference
Fracture risk assessment and case finding	Bai et al 2020	MA	Low	[71]
	Gausden et al 2017	SR	Medium	[106]
	Johannesdottir et al 2018	SR	Low	[54]
	Kanis et al 2016	SR	Medium	[90]
	Marshall et al 1996	MA	Critically Low	[40]
	Merlijn et al 2019	SR & MA	Critically Low	[113]
	Mortensen et al 2020	SR & MA	Medium	[77]
	Singh-Ospina et al 2017	SR & MA	Low	[84]
	Vilaca et al 2020	SR & MA	Low	[70]
	Zhang et al 2020	SR & MA	Low	[115]
Intervention thresholds and management strategy	Kanis et al 2016	SR	Medium	[90]
Non-pharmacological management of osteoporosis	Babatunde et al 2020	SR & MA	Medium	[169]
	El-Khoury et al 2013	SR & MA	Medium	[175]
	Darling et al 2019	SR & MA	Medium	[153]
	Fabiani et al 2019	SR & MA	Medium	[150]
	Gillespie et al 2012	SR & MA	High	[179]
	Groenendijk et al 2019	SR & MA	Medium	[152]
	Iguacel et al 2018	SR & MA	High	[155]
	Howe et al 2011	SR & MA	High	[167]
	Jepsen et al 2017	SR & MA	Medium	[180]
	Kahwati et al 2018	SR & MA	Medium	[164]
	Kelley et al 2000	SR & MA	Medium	[170]
	Kemmler et al 2020	SR & MA	Low	[168]
	Kunutsor et al 2018	SR & MA	Medium	[172]
	Min et al 2017	SR & MA	Low	[183]
	Shen et al 2015	SR & MA	Medium	[182]
	Sherrington et al 2017	SR & MA	Low	[178]
	Sherrington et al 2019	SR & MA	High	[176]
	Yao et al 2019	SR & MA	Medium	[160]
	Zhao et al 2019	SR & MA	Low	[177]
Pharmacological treatment options	Beaudart et al 2025	SR & MA	Critically Low	[188, 241]
	Diez-Perez et al 2019	SR & MA	Medium	[241]
	Gartlehner et al 2017	SR & MA	Medium	[330]
	Nayak et al 2017	SR & MA	Low	[331]
	Poon et al 2018	SR & MA	Low	[290]
	Simpson et al 2020	SR & MA	Medium	[242]
	Zeng et al 2019	SR & MA	Medium	[332]
Strategies for management of osteoporosis and fracture risk	Deng et al 2020	SR & MA	Low	[283]
	Dennison et al 2019	SR	Medium	[215]
	Gedmintas et al 2013	SR & MA	Medium	[276]
	Khan et al 2015	SR	Medium	[269]
	Miyashita et al 2020	SR & MA	Low	[295]
	Nayak et al 2019	SR & MA	High	[258]
	Tsourdi et al 2020	SR	Medium	[213]
	Wang et al 2018	SR & MA	Critically Low	[333]
	Yanbeiy et al 2019	SR & MA	Low	[334]
Management of symptomatic osteoporotic vertebral fractures	Al-Sari et al 2016	SR & MA	Low	[14]
	British Geriatric Society 2013	SR	Medium	[298]
	Buchbinder et al 2018	SR & MA	High	[306]
	Ebeling et al 2019	SR & MA	Critically Low	[303]
	Gibbs et al 2019	SR	Medium	[302]
	Hofler et al 2020	SR	Low	[305]
	Knopp-Sihota et al 2012	SR & MA	Medium	[299]
	Svensson et al 2017	SR	Low	[304]
Models of care for fracture prevention	Ganda et al 2013	SR & MA	Critically Low	[314]
	Ganda et al 2019	SR & MA	Low	[315]
	Martin et al 2020	SR & MA	Medium	[323]
	Paskins et al 2020	SR	Medium	[335]
	Wu et al 2018	SR	Critically Low	[309]
	Wu et al 2018	SR & MA	Low	[316]

*SR*, Systematic Review. *MA*, Meta-analysis
